# Resolving the Fast Kinetics of Cooperative Binding: Ca^2+^ Buffering by Calretinin 

**DOI:** 10.1371/journal.pbio.0050311

**Published:** 2007-11-27

**Authors:** Guido C Faas, Beat Schwaller, Julio L Vergara, Istvan Mody

**Affiliations:** 1 Department of Neurology, University of California Los Angeles, Los Angeles, California, United States of America; 2 Unit of Anatomy, Department of Medicine, Universitaet Fribourg, Fribourg, Switzerland; 3 Department of Physiology, University of California Los Angeles, Los Angeles, California, United States of America; University of Texas Austin, United States of America

## Abstract

Cooperativity is one of the most important properties of molecular interactions in biological systems. It is the ability to influence ligand binding at one site of a macromolecule by previous ligand binding at another site of the same molecule. As a consequence, the affinity of the macromolecule for the ligand is either decreased (negative cooperativity) or increased (positive cooperativity). Over the last 100 years, O_2_ binding to hemoglobin has served as the paradigm for cooperative ligand binding and allosteric modulation, and four practical models were developed to quantitatively describe the mechanism: the Hill, the Adair-Klotz, the Monod-Wyman-Changeux, and the Koshland-Némethy-Filmer models. The predictions of these models apply under static conditions when the binding reactions are at equilibrium. However, in a physiological setting, e.g., inside a cell, the timing and dynamics of the binding events are essential. Hence, it is necessary to determine the dynamic properties of cooperative binding to fully understand the physiological implications of cooperativity. To date, the Monod-Wyman-Changeux model was applied to determine the kinetics of cooperative binding to biologically active molecules. In this model, cooperativity is established by postulating two allosteric isoforms with different binding properties. However, these studies were limited to special cases, where transition rates between allosteric isoforms are much slower than the binding rates or where binding and unbinding rates could be measured independently. For all other cases, the complex mathematical description precludes straightforward interpretations. Here, we report on calculating for the first time the fast dynamics of a cooperative binding process, the binding of Ca^2+^ to calretinin. Calretinin is a Ca^2+^-binding protein with four cooperative binding sites and one independent binding site. The Ca^2+^ binding to calretinin was assessed by measuring the decay of free Ca^2+^ using a fast fluorescent Ca^2+^ indicator following rapid (<50-μs rise time) Ca^2+^ concentration jumps induced by uncaging Ca^2+^ from DM-nitrophen. To unravel the kinetics of cooperative binding, we devised several approaches based on known cooperative binding models, resulting in a novel and relatively simple model. This model revealed unexpected and highly specific nonlinear properties of cellular Ca^2+^ regulation by calretinin. The association rate of Ca^2+^ with calretinin speeds up as the free Ca^2+^ concentration increases from cytoplasmic resting conditions (∼100 nM) to approximately 1 μM. As a consequence, the Ca^2+^ buffering speed of calretinin highly depends on the prevailing Ca^2+^ concentration prior to a perturbation. In addition to providing a novel mode of action of cellular Ca^2+^ buffering, our model extends the analysis of cooperativity beyond the static steady-state condition, providing a powerful tool for the investigation of the dynamics and functional significance of cooperative binding in general.

## Author Summary

The binding of a ligand to a protein is one of the most important steps in determining the function of these two interactive biological partners. In many cases, successive binding steps occur at multiple sites such that binding at one site influences ligand binding at other sites. This concept is called cooperative binding, and constitutes one of the most fundamental properties of biological interactions. The functional consequences of cooperativity can be accurately resolved when reactions are at equilibrium, but mathematical complexity has prevented insights into the dynamics of the interactions. We studied the protein calretinin, which binds Ca^2+^ in a cooperative manner and plays an important role in shaping Ca^2+^ signals in various cells. We used two models, a widely tested one and a novel, mathematically simplified one, to resolve the dynamics of a cooperative binding process. The cooperative nature of Ca^2+^ binding to calretinin results in accelerated binding as calretinin binds more Ca^2+^. This behavior constitutes an important new insight into the regulation of intracellular Ca^2+^ that cannot be matched by noncooperative artificial Ca^2+^ buffers. Our simple mathematical model can be used as a tool in determining the kinetics of other biologically important molecular interactions.

## Abstract 3

A novel and relatively simple mathematical model for the kinetics of cooperative binding reveals, the tuning of Ca^2+^-buffering kinetics due to cooperative binding in calretinin.

## Introduction

In all eukaryotic cells, Ca^2+^ signals play a crucial role in the regulation of many cellular processes, including gene expression, cytoskeleton dynamics, cell cycle, cell death, neurotransmission, and signal transduction. To achieve its role as messenger, the intracellular Ca^2+^ concentration ([Ca^2+^]) is very tightly regulated in time, space, and magnitude. The spatiotemporal characteristics of short-lived and often highly localized changes in intracellular [Ca^2+^] result from a complex interplay between Ca^2+^ influx/extrusion systems, mobile/stationary Ca^2+^-binding proteins (CaBPs), and intracellular sequestering mechanisms. Understanding the kinetics of cellular Ca^2+^ transients and its influence on Ca^2+^-regulated processes requires a precise knowledge of the Ca^2+^ sensitivities and binding properties of all the components involved, including the binding dynamics to buffering and signaling CaBPs. However, uncertainties in current models studying intracellular Ca^2+^ signaling arise mostly from the lack of accurate data on the binding properties of specific molecules involved in Ca^2+^ handling, considerably limiting the value of such modeling [1]. An important step towards the goal of precisely describing intracellular Ca^2+^ transients was the study by Nagerl et al. [2], in which the relevant parameters (affinities and on- and off-rates of Ca^2+^ binding) for the CaBP calbindin D-28k (CB) were determined in vitro by flash photolysis of caged Ca^2+^. Cooperative binding of Ca^2+^, known to play a significant role in multisite CaBPs such as calmodulin [3] and calretinin (CR) [4], has never been directly determined in rapid kinetic experiments, but only inferred from steady-state conditions using Hill [5] and Adair-Klotz models [6,7].

Cooperativity first evidenced by oxygen binding to hemoglobin [8] is considered one of the most imperative functional properties of molecular interactions in biological systems, even considered to be the great secret of life, second only to the structure of DNA [9]. Cooperativity is the ability to influence ligand binding at a site of a macromolecule by previous ligand binding to another site of the same macromolecule. Many proteins show increased (positive cooperativity) or decreased (negative cooperativity) affinity for a ligand after binding of a first ligand. Over the last 100 years, hemoglobin has been a paradigm for cooperative ligand binding and allostery. Oxygen binding to hemoglobin resulted in four commonly used descriptions for cooperativity (for review see [10]): the Hill [11], the Adair-Klotz [6,7], the Monod-Wyman-Changeux (MWC) [12], and the Koshlan-Némethy-Filmer (KNF) [13] models. Yet all these models describe cooperativity only when the binding reactions are at equilibrium. Since temporal aspects of most ligand binding processes are essential for correct physiological functioning, it is imperative to consider the kinetics of cooperative binding. To date, studies determining the kinetics of cooperative binding to biologically active molecules have been carried out using the MWC model, in which cooperativity is established by assuming two allosteric isoforms with different binding properties. These studies were limited to special cases where transition rates between allosteric isoforms are much slower than the binding rates [14,15] or where binding and unbinding rates could be measured independently [16]. For all other cases, the mathematical description becomes too complex for simple interpretations [10,17]. The Hill equation is perhaps the oldest and most widely used description for the relative amount of binding by a cooperative molecule, and the cooperative binding is described with two constants: the dissociation constant (*K*_d_) reports on the concentration of ligand at which the cooperative molecule is half occupied and the Hill number (*n*_H_) describes the steepness of the binding curve at the value of *K*_d_, denoting a simple quantification of cooperativity. Although not representing a mathematically correct description of cooperative binding at equilibrium—a fact that is stated in the original work [11]—the Hill equation has proven to be extremely useful, as it describes occupancy as a function of ligand concentration with merely two constants that are easy to interpret intuitively. With this in mind, we wanted to resolve the kinetics of Ca^2+^ binding to CR and to find an intuitively “accessible” quantitative description of the binding kinetics.

CR belongs to the superfamily of EF-hand Ca^2+^-binding proteins. This superfamily is named after the common Ca^2+^ binding structure—the EF-hand—first described as the C-terminal E-helix–loop-F-helix Ca^2+^ binding site in parvalbumin [18]. Most members have an even number of EF-hand domains organized in pairs [19], representing a structurally conserved architectural unit. CR has six EF-hand domains [4,20–24], which can be subdivided into two independent domains: one with the cooperative pair of binding sites I and II, and another with binding sites III–VI [23]. Sites III–VI can be further subdivided into one cooperative pair, sites III and IV and sites V and VI [4]. Of the latter pair, only site V binds Ca^2+^, whereas site VI is “inactive” [4]. Thus, CR has two pairs of cooperative binding sites (I–II and III–IV) and one independent binding site (V) for Ca^2+^. We tried several approaches to describe the kinetics of these five binding sites based on the published models, and discovered a new and simplified kinetic model that quantitatively resolves the kinetics of cooperative binding. This new model also revealed unexpected and highly specific nonlinear properties of cellular Ca^2+^ regulation by CR.

## Results

### The Binding Sites of Calretinin

**Figure 1:**
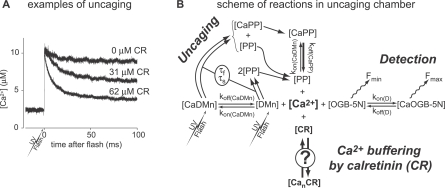
Ca^2+^ Measurements In Vitro (A) Examples of changes in free [Ca^2+^] after photolysis of DMn in the absence of protein (upper trace) and in the presence of 31 μM and 62 μM CR (middle and lower traces, respectively). The UV flash energies used to uncage DMn were of similar magnitude, resulting in an equivalent amount of uncaged Ca^2+^. (B) Scheme of all equilibrium reactions occurring in the measurement chamber after photolysis of caged Ca^2+^. The kinetic rate constants for DMn, its photoproducts (PP), and the Oregon Green BAPTA 5N (OGB-5N), and uncaging time constants (τ_f_ and τ_s_) of DMn were independently determined. The reaction parameters to be determined for describing the Ca^2+^ binding to CR are indicated by a question mark (**?**).

We determined the kinetics of Ca^2+^ binding to CR by Ca^2+^ uncaging using DM-nitrophen (DMn) and measuring the changes in [Ca^2+^] with the fluorescent Ca^2+^ indicator dye Oregon Green BAPTA-5N (OGB-5N) as previously described [2,25]. Changes in the OGB-5N fluorescence were observed immediately after photolysis of DMn in solutions containing various concentrations of CR (Figure 1A). A rapid rise in [Ca^2+^] ensued from a resting concentration of approximately 2.4 μM to 11 μM. At this initial [Ca^2+^], approximately 99.5% of the DMn is in the Ca^2+^-bound form, ensuring that (1) virtually every uncaged DMn molecule will release Ca^2+^, and (2) the amount of free DMn capable of rebinding uncaged Ca^2+^ ions [25,26] is considerably limited. This is evidenced by the negligible drop in [Ca^2+^] in the absence of CR, leading to an almost step-wise increase in [Ca^2+^] (Figure 1A). The presence of CR (31 and 62 μM) resulted in a [CR]-dependent drop in [Ca^2+^]. Figure 1B depicts a simplified reaction scheme of the experiment. To determine the association and dissociation rates of Ca^2+^ binding to CR, all these different reactions were incorporated into a mathematical model (see below). The aim was to find a mathematical description for the Ca^2+^-binding properties of CR that best fits the experimental fluorescence traces generated under various conditions.

As a starting point to determine CR's kinetics of Ca^2+^ binding, we relied on steady-state Ca^2+^-binding properties determined previously. With the same human recombinant CR, selected Hummel-Dryer experiments yielded a Hill coefficient of 1.3 for the four binding sites, with a *K*_d_ of 1.5 μM [4]. However, by flow dialysis, the steady-state binding of Ca^2+^ to human CR could be described with the following macroscopic constants (*K*_1_ through *K*_5_) 2.2 × 10^5^ M^−1^, 3.2 × 10^5^ M^−1^, 4.7 × 10^5^ M^−1^, 8.0 × 10^5^ M^−1^, and 2.0 × 10^4^ M^−1^ [4]. The resulting binding curve derived from these values could be accurately fitted with two Hill equations; one equation described four cooperative binding sites with a *K*_d_ of 2.5 μM and a Hill coefficient of 2.4, and the other one described a single independent site with a *K*_d_ of 53 μM. In agreement with this data, equilibrium dialysis experiments with chick CR revealed a Hill coefficient of 1.9 [22]. Even Hill coefficient values of up to 3.7 for Ca^2+^-induced tryptophan (Trp) fluorescence changes in rat CR have been reported [21]. However, these conformational changes measured by Trp fluorescence do probably not linearly relate to Ca^2+^ binding. Thus, the absolute values should be interpreted with caution, but nonetheless, cooperativity of Ca^2+^ binding also occurs in rat CR. Based on these Hill coefficients of 1.3, 1.9, and 2.4, we conjectured that CR has four binding sites for Ca^2+^ with positive cooperativity, with a Hill coefficient of approximately 2 and one independent binding site for Ca^2+^.

In accordance with these and other earlier findings on the structure and physiology of CR (see Introduction), we modeled the protein as possessing two pairs of cooperative binding sites (B_I_B_II_) and (B_III_B_IV_), and one independent binding site B_V_. Because the properties of the two cooperative pairs in CR were considered indistinguishable in the steady-state study [4], thus indicating that the cooperative binding sites are fairly similar, we assumed the properties of both cooperative pairs to be identical:

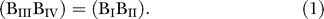



Such an assumption is most useful for reducing the number of variables, thus increasing the reliability of fitting procedures by constraining the model.

### Analysis of Ca^2+^ Binding to CR Using a New Model That Includes Cooperativity

**Figure 2:**
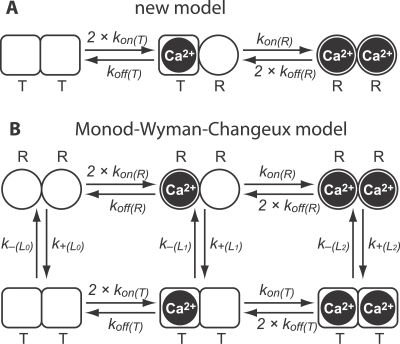
Models Used to Simulate Cooperativity Both models used to simulate cooperativity are based on the ability of the binding sites to occur in two states, one with a low affinity for Ca^2+^ (T) and one with a high affinity (R). In the new model (A), a binding site is in the T state when the other binding site has no Ca^2+^ bound, and it is in the R state when the other site has Ca^2+^ bound. In this model, the transitions from T to R and vice versa are considered to occur instantaneously. In the MWC model, the cooperative pair of binding sites are either both in the T state or both in the R state. The transitions between R and T occur according to the transition constants *k*_+_ and *k*_−_. For each occupation level, there is a separate equilibrium (*L*_0_, *L*_1_, and *L*_2_) for which it can be shown that under specific conditions, the equilibrium shifts from mainly the T state in the unoccupied cooperative sets to mainly the R state in fully occupied states (see also Equations 8, 9, and 10).

The most straightforward approach to determine the kinetics of a system would consist of fitting the [Ca^2+^] decay with a set of exponential functions. However, rebinding of Ca^2+^ to free DMn affects the decay kinetics. In addition, the changing properties of the binding sites that underlie cooperativity are expected to cause a shift in the kinetic properties of binding during the decay phase. As a consequence, the relative contribution of multiple decay time constants is continuously shifting. Although fitting the [Ca^2+^] decay with exponentials might result in time constants for a given trace, this does not allow accurate deduction of the Ca^2+^-binding kinetics of CR. A mathematical model simultaneously describing all processes taking place in the recording chamber, including a “total” description of the cooperative and noncooperative binding of Ca^2+^ to CR is expected to yield more reliable information on the kinetic properties of CR. To model the cooperativity, we started out by including an allosteric influence between the binding sites of the pairs. This was achieved by setting two states (R and T) for a particular binding site, with each having its own set of rate constants. A binding site is in the “tensed” state (T), with a low affinity for Ca^2+^, when no Ca^2+^ is bound to the other site in the pair, whereas a binding site is in the “relaxed” state (R), with a high affinity for Ca^2+^, when the other site already has a Ca^2+^ ion bound. We assumed that binding of Ca^2+^ to one site always leads to a rapid transition T→R in the other site and that an unbinding of Ca^2+^ from one site always leads to a rapid transition R→T in the other site. This allowed us to incorporate the transition rates between states R and T in the binding and unbinding rate constants, further simplifying the model (Figure 2A). CR was thus modeled as if consisting of two independent proteins described by the following binding reactions:

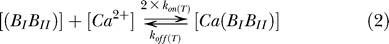


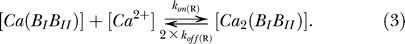



This cooperative part of the model can be easily related (see Discussion) to the Adair model [6], which provides the most general description of equilibria in terms of stochiometric binding. For the independent site of CR, we used a standard equilibrium equation:

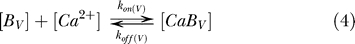
where *k*_on(R or T)_ and *k*_off(R or T)_ are the association and dissociation rate constants for the individual cooperative binding sites depending on their Ca^2+^-binding status, and *k*_on(V)_ and *k*_off(V)_ are the rate constant for the independent site. The total concentrations of the different “virtual” parts are:

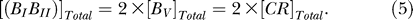



**Figure 3:**
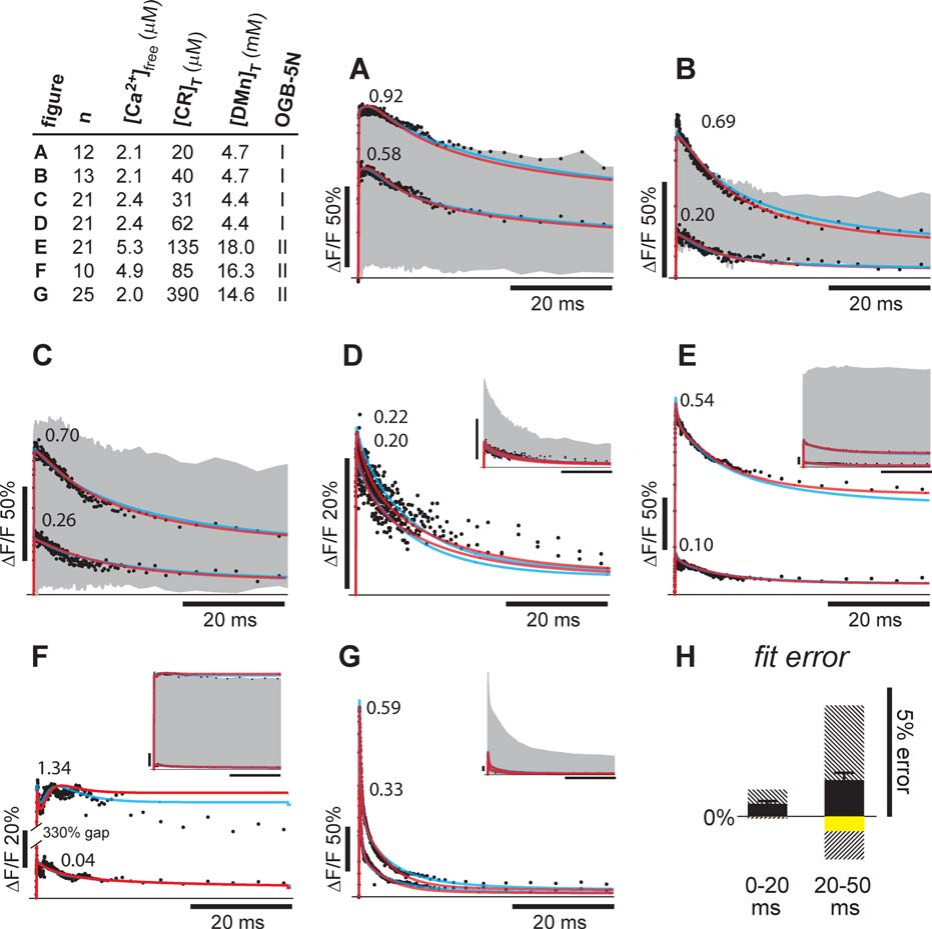
Fitting of the Ca^2+^ Uncaging Data with the Model of Two Pairs of Cooperative Sites and One Individual Site A typical dataset (•) consisting of 14 randomly chosen traces is shown in (A–G). The traces were selected from seven different experimental conditions, two per condition. The table shows the values for the four variables differing in conditions (A–G). Individual data points (•) are fitted with either the new model (red lines) or the MWC model (blue lines), taking into consideration the four variables listed in the table, whereas the values for *k*_on_'s, *k*_off_'s, *K*_d(app)_, *L*_0_, *k*_+(L0)_, and the *n*_H_ for CR are identical for all fits within a set. The amount of uncaging is fitted independently for every trace. For the selected examples, it is expressed as percentage of total [DMn]. The gray areas indicate the whole range of uncaging experiments for each experimental condition; the smaller insets in (D–G) show the whole range of traces, whereas the *y*-axis ranges in the main panels (D–G) were selected to optimally show the 14 selected traces. More individual curves can be seen in Figure S1. Black scale bars in (A–G) insets indicate 50% Δ*F*/*F* (*y*-axis) or 20 ms (*x*-axis). (H) To assess the goodness of fit by the new model, the averaged relative deviations of the fit from the data are shown as black bars ± the standard error of the mean for the first 20 ms of the fit (left) and for 20–50 ms of the fit (right bar). The largest single deviations in either direction found in all of the sets are indicated by striped bars. The yellow bars indicate the deviation observed in the selected set of traces displayed in (A–G). The scale bar indicates 5% error/deviation from data. In the table for the OGB-5N column, I refers to lot number 34B1–2 and II to lot 15C1–2.

**Figure 4:**
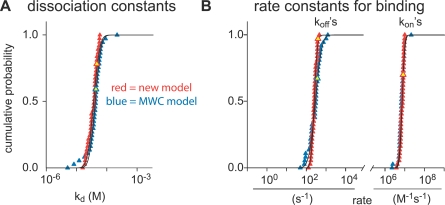
Properties of the Independent Site The 38 fit results for the *k*_on_'s, *k*_off_'s (B), and *K*_d_'s (A) of the independent site (V) have a log-normal distribution for both models. This can be seen when the data of the new model (red symbols) and the MWC model (blue symbols) are plotted on a logarithmic scale as a cumulative probability plot. The *k*_on_'s, *k*_off_'s (B), and, *k*_d_'s (A) were fitted (solid lines) with a single log-normal function, indicating a uniform result for the 38 fits. The yellow symbols are derived from the dataset shown in Figure 3.

**Figure 5:**
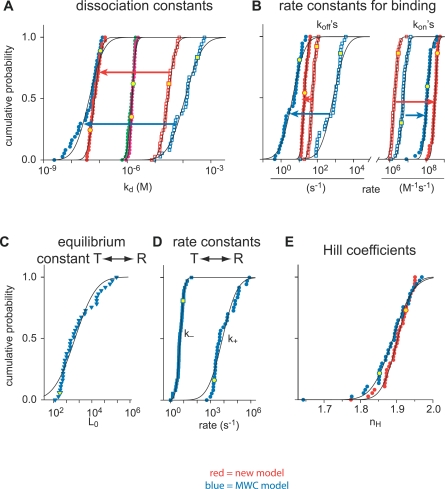
Properties of the Cooperative Sites The data of the 38 fit results for the rate and affinity constants have a log-normal distribution, evidenced when plotted on a logarithmic scale as a cumulative probability (A–D). All data could be fitted (solid lines) with a single log-normal function, indicating a unique result for the 38 fits. The Hill coefficient for both models was normally distributed (E) and was fitted accordingly. In all subfigures (A–E), red symbols indicate results for fits with the new model and blue symbols indicate results for the fits with the MWC model. The values for the dissociation constants in (A) and the rate constants in (B) found for the T state are indicated by open squares (□), whereas the values for the R state are indicated with closed circles (•). The arrows indicate the general shift from T state to R state as the cooperative sites bind Ca^2+^. The apparent dissociation constants (A) are indicated with pink circles for the new model and green circles for the MWC model. The equilibrium constants and rate constants for the transition from the T to the R state in the MWC model are shown in (C) and (D), respectively. The Hill coefficients obtained are shown in (E). The yellow symbols are the data points derived from the experiment shown in Figure 3.

Despite the simplifying assumptions concerning the cooperative sites, the model that allows a fitting routine to proceed is fairly complex, because it has a considerable number of degrees of freedom. Thus, a procedure was developed that significantly constrains the fit to minimize the variance of the fit results. Simultaneously fitting combined sets of uncaging data (Figure 3; see Materials and Methods) obtained under different experimental conditions sufficiently constrained the model to yield consistent results. We performed a number of individual uncaging experiments generated at one of seven initial conditions A–G (Table in Figure 3). These conditions varied in the initial free [Ca^2+^] (hence, total [Ca^2+^]), total [CR], total [DMn], as well as on the lot number of OGB-5N, with each lot having slightly different properties (see Materials and Methods). Under each condition (A–G), we performed 12 to 25 uncaging experiments, each one with a different flash energy of the UV laser leading to different amounts of uncaged Ca^2+^. In total, 123 traces were obtained, covering a wide rage of uncaging energies and, subsequently, a wide range of increases in [Ca^2+^] (see gray areas in Figure 3; for all 123 individual traces, see Figure S1). We set out to find a satisfactory model that would be able to fit all curves obtained under conditions A–G and all tested uncaging intensities. The obtained results from the modeling should be able to describe all experimental curves with a unique set of parameters describing the kinetic properties of CR. To confine the fits, 38 sets of 14 pseudo-randomly picked traces consisting of two traces from each initial condition A–G were generated. The 38 sets were chosen randomly, with the precondition that every trace of a specific starting condition A–G was represented equally. To create 38 sets, each individual measurement was picked at least three and at most eight times. On average, each trace was picked 4.3 times (for details, see Figure S2). Each set of traces was fitted with the model, and the fitted parameters describing the properties of CR were constrained to be identical for all individual traces within one set. The only variable parameter between traces was the amount of uncaging that was fitted individually for each trace. An example of a dataset of 14 traces (two sets of data points [•] for each condition [A–G]) and the fitted traces (red or blue lines, see Figures S1–S3 for additional details) are shown in Figure 3. Fit results for this dataset are depicted in Figures 4 and 5 (yellow symbols) together with the results of the fits on the other 37 sets.

The new model was programmed as to fit the *K*_d(V)_ and *k*_on(V)_ for the independent site. However, to aid the choice of starting values, the cooperative part of the model was set up such that *k*_on(R)_, *k*_on(T)_, the apparent *K*_d_ (*K*_d(app)_) for the pairs, and the Hill number (*n*_H_) could be fitted. This was achieved by adding a calculation step that determined *K*_d(R)_ and *K*_d(T)_ from the latter two parameters (see Protocol S1):

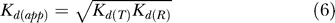


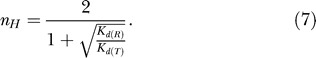



Previously determined steady-state parameters (apparent) *K*_d_'s and *n*_H_ of CR [4,20–22] served as starting points in the modeling and helped to further constrain the model. Various combinations of *k*_on_ starting values between 10^5^ and 10^8^ M^−1^s^−1^ were tested, but this did not significantly influence the outcome of the fit, indicative of the “robustness” of the modeling procedure. Occasionally a particular set out of the 38 yielded an atypical fit with values significantly deviating from the general population of results. If this was the case, we followed up with two approaches. First, we tested whether any of the other 37 sets of fluorescence traces could also be fitted with these deviating values, which in almost all cases yielded unsatisfactory fits. Second, we tested whether the deviating set of traces could also be fitted with the more homogeneous values of the general population of sets by choosing starting parameters closer to these values. In this case, the deviating set could always be fitted with values comparable to the homogeneous constants. It should be noted that the critical parameters (*K*_d(V),_ *k*_on(V),_ *k*_on(R)_, *k*_on(T)_, *K*_d(app)_, and *n*_H_) were never constrained or fixed to a certain value. The atypical fit results were probably caused by local minima in the error function of the fit routine. Such local minima are expected, based on the fairly large number of degrees of freedom where the parameters are not completely independent. Deviations in one parameter can be partially compensated by “shifting” other parameters. Initially, we used a model that did not include cooperativity and found that most of the 38 individual sets could be fit reasonably well. For a given individual dataset, the quality of the fits were similar between a model with a Hill coefficient of either 1 or 1.9. But when comparing the fit results of all 38 sets, most of the fitted parameters showed strong deviations (up to five orders of magnitude, depending on how the model was exactly defined) when using *n*_H_ = 1. The high variability of the binding parameters found when assuming *n*_H_ = 1 is shown in Figure S3. This indicated that there is no unique solution to describe CR's Ca^2+^-binding properties without cooperativity, in line with previous steady-state findings of *n*_H_ values between 1.3 and 2.4 [4,22]. Thus, only when including cooperativity and starting the modeling procedure with previously determined steady-state parameters for CR [4,20–22] did we find a congruent set of values for the fitted parameters for all 38 sets of 14 traces.

An accurate model describing CR's Ca^2+^-binding dynamics should be able to fit all the experimental traces obtained under any condition. This should be the case at lower resting [Ca^2+^], when Ca^2+^-free binding sites of any affinity in any state are abundant (Figure 3A–3D and 3G), but also at higher [Ca^2+^], when mostly the lower affinity independent site V is available (Figure 3E and 3F). Furthermore, the model is also able to closely describe the [Ca^2+^] signals after a relatively large uncaging, when [Ca^2+^] is so high that the buffering by CR is relatively small (see upper trace in Figure 3F). The goodness of fit of the fit procedure can be appreciated by the averaged error for the 38 fits (Figure 3H, black bars), which shows systematic deviations (if any). Errors were found to be extremely small for the first 20 ms after the flash, and at time points greater than 20 ms, the averaged fits show a small systematic undershoot of the experimental data, yet never exceeding 1.5% of the actual amplitude (Figure 3H, black bars). The larger errors towards the end of the traces are likely due to the small amplitudes of the signals at these time points, which increase the relative error when there is a constant absolute deviation. But even the largest deviations of the 38 fits (the striped bars showing the largest deviation in either direction) never exceeded 5% of the measured amplitude. The average absolute error (not allowing for positive and negative errors to cancel each other) was maximally 2.1% and again only found towards the end of the traces. Thus, the fit procedure, applying our model, allowed accurate quantifying of the kinetic properties of CR.

All 38 results for the fitted values were plotted as a log-normal cumulative probability distribution because they have a log-normal distribution (Figures 4 and 5), except for the *n*_H_ value, which was normally distributed (Figure 5E). These results were then fitted with a normal distribution to determine average and standard deviation. The results of these fits are shown in Table 1. To summarize, we conclude that CR can be described with one independent binding site with a *K*_d_ of 36 μM, a *k*_on_ of 7.3×10^6^ M^−1^s^−1^, and a *k*_off_ of 240 s^−1^ together with two identical cooperative pairs of binding sites with an initial (T state) *K*_d_ of 28 μM with a *k*_on_ of 1.8×10^6^ M^−1^s^−1^ and a *k*_off_ of 53 s^−1^ that will dramatically change to a (R state) *K*_d_ of 68 nM with a *k*_on_ of 3.1 × 10^8^ M^−1^s^−1^ and a *k*_off_ of 20 s^−1^ once the cooperative partner site has already bound Ca^2+^. The *K*_d(app)_ for the cooperative sites is 1.4 μM with an *n*_H_ of 1.9.

**Table 1:**
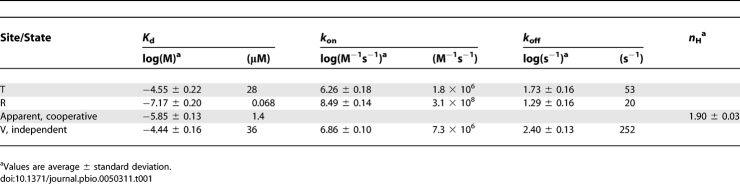
Results for the New Model (by Fitting the Results of the 38 Fit Sets)

### Modeling Cooperativity of Ca^2+^ Binding to CR Using a Monod-Wyman-Changeux Model

To compare the results obtained with our new “simple” model, we subjected our experimental data to a previously described cooperative model, the MWC model. There, the protein as a whole can switch between two states: one state in which all the binding sites are in the T state, and another one in which all the binding sites are in the R state (Figure 2B). The equilibrium between the T and R states is described with the equilibrium constant *L*, which is also dependent on the number of Ca^2+^ ions bound (see Figure 2B). It can be shown [12,27] that:

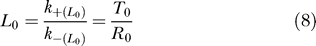


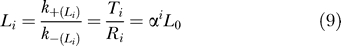


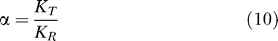
where *k*_+_ and *k*_−_ are rates of the transition between the two states R and T. The indices (0 and *i* in Equations 8 and 9, respectively) indicate the number of Ca^2+^ ions bound. *K* is often indiscriminately used for both association and dissociation equilibrium constants; here, we denote *K* as association equilibrium constants, whereas we use *K*_d_ for dissociation equilibrium constants. Furthermore, the pair of cooperative binding sites can transition from R to T and back, independently of the number of sites that are occupied, thus all transitions defined by *L*_0_, *L*_1_, and *L*_2_ are possible. However, for steady-state purposes, one transition (*L*_0_) suffices, as described in the original MWC model [12]. Although steady-state properties are independent of the transitions allowed, the kinetic properties will highly depend on the number of allowed transitions. We chose to allow all possible transitions because it was used in earlier kinetic fits with this model [14,16]. We also attempted to fit the data with a MWC model in which only the *L*_0_ transition was possible; however, the resulting fits showed large deviations (>20%) and generated traces with significant deviations from the experimental data. From Equation 9, we can derive the information that while *K*_T_ < < *K*_R_ and *L*_0_ > > 1, the equilibrium between the T and R states is shifted towards the lower affinity T state when no or little Ca^2+^ is bound, whereas it shifts towards the higher affinity R state when plenty of Ca^2+^ is bound [27], which causes the cooperative effect.

The identical 38 sets of 14 experimental traces as used above were fitted with the MWC model. Here also, both cooperative sets were considered to be identical and the cooperative part of the model was set up such that *k*_on(R)_, *k*_on(T)_, the apparent *K*_d_ (*K*_d(app)_) for the pairs, and the Hill number (*n*_H_), could be fitted. This was achieved by adding a calculation step that determined *K*_d(R)_ and *K*_d(T)_ from the latter two parameters (see Protocol S1):

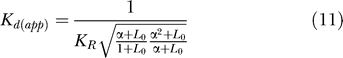


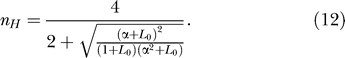



As discussed above, *L* is dependent on the number of Ca^2+^ ions bound to CR. To establish this dependence, we changed the forward and backward rate constants between the R and T states equally:

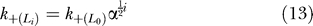


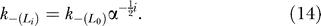



We started with the same values for the (apparent) *K*_d_'s and *n*_H_ as above with various combinations of *k*_on_ starting values between 10^5^ and 10^8^ M^−1^s^−1^. As with the first model, occasionally a set of traces was fitted with values that deviated significantly from the general population, but again we found that only with one general set of constants, all 38 sets could be accurately fitted; the details are reported in Table 2. For comparison, we depicted the fit and the fit errors obtained with the MWC model, using the identical set of traces used for the new model (compare Figure 3A–3G, blue vs. red traces; errors for MWC fit are not shown, but are comparable to the fits with the other model), and observed that the MWC model can fit the data with a similar accuracy as our new model. The average results from all traces were obtained as described for the new model (Figures 4 and 5). For the independent site (Figure 4A and 4B), results based on the MWC model (blue symbols) were essentially identical to the ones found with the new model (red symbols, compare also Tables 1 and 2 for the independent site V). Also, the properties of the apparent *K*_d_ and the *n*_H_ of the cooperative sites were similar between the two models (Figure 5A, compare green and pink circles, and Figure 5E, compare blue and red symbols). This is a first indication that both models quantitatively describe the same process. Obviously, the detailed descriptions for the cooperative sites, applying either the new or the MWC model, deviate from one another, based on the differently modeled processes as described in Figure 2.

**Table 2:**
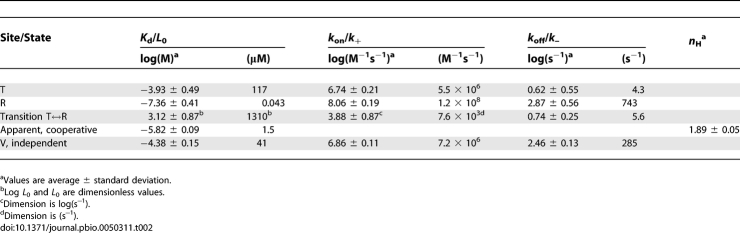
Results for the MWC Model (by Fitting the Results of the 38 Fit Sets)

### Comparison of the Kinetic Properties of CR Determined by Either the New or the MWC Model

**Figure 6:**
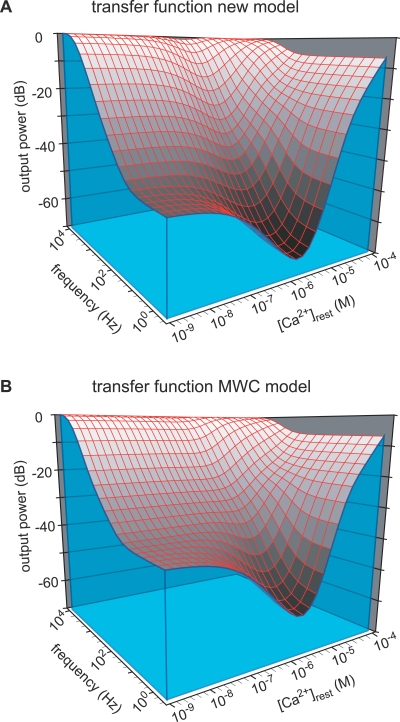
Transfer Function of CR The transfer functions for 500 μM CR simulated with either the new model (A) or the MWC model (B). The transfer functions for 500 μM CR were determined by simulating a 1 nM fluctuation in [Ca^2+^] at different frequencies and various resting [Ca^2+^]. The output was determined from the peak-to-peak amplitude of the resulting Ca^2+^ waveform.

Both models accurately fitted the data in a quantitative manner; based on the quality of the fits, they were indistinguishable, and technically can both be used to quantify the kinetic properties of Ca^2+^ binding to CR. In particular, results for the independent site (V) are virtually identical (Figure 4). For the cooperative sites, both models describe binding sites that have a similar steady-state/affinity profile (Figure 5A; apparent *K*_d_ and Figure 5E; *n*_H_). Our uncaging experiments were performed over a fairly narrow range of resting [Ca^2+^] (2.0–5.3 μM), dictated by the constraints that most (>99%) of the DMn should be in the Ca^2+^-bound form to obtain valuable data. At lower resting [Ca^2+^], the unbound DMn, present at higher concentrations than the free [CR], would rapidly rebind most of the released Ca^2+^ [25], making CR's relative contribution to the [Ca^2+^] decay small and difficult to distinguish. Because buffering kinetics depend on both the on-rates and the concentration of free buffer, the overall Ca^2+^ binding speed to DMn would be much higher than that to CR, thus masking Ca^2+^ binding to CR. With much less Ca^2+^-bound DMn, the changes in [Ca^2+^] would be quite small and difficult to detect. Such technical constraints do not allow performing the experiments over the whole “physiological” range of [Ca^2+^], e.g., from 10 nM to 100 μM. Thus, we could not exclude that the two models describe systems with different kinetic properties outside the boundaries of our experiments. This possibility was tested by examining the behavior of each model as a filtering system for Ca^2+^ signals regulated by resting levels of [Ca^2+^]. The filtering properties of both models were determined over a range of conditions covering the whole physiological range that CR is expected to encounter. CR (500 μM) was subjected to a wide frequency range (0.3 Hz to 10 kHz) of small (1 nM) sinusoidal perturbations of the [Ca^2+^] at a wide range of starting [Ca^2+^] (1 nM to 100 μM). The resulting Ca^2+^ “waves” were close to sinusoidal. We used their amplitude as output of the filter to determine the transfer function of CR (Figure 6). The attenuation of the sine wave is plotted as a function of frequency and resting [Ca^2+^]. Both models “filter” Ca^2+^ signals in a very similar way; the signals are less attenuated as the frequency gets higher (CR acts as a high-pass filter) or when CR becomes fully occupied as the starting [Ca^2+^] gets higher. Remarkably, both models show a similar strong increase in attenuation at the lower frequencies, when the Ca^2+^ concentration gets close to the apparent *K*_d_ value of the cooperative sites, i.e., approximately 1.5 μM. The similarity of the transfer function of CR using either model indicates that they quantify the kinetics of Ca^2+^ binding by CR in a similar way (but via different mechanisms) over the whole physiological range of conditions.

### Comparing the New Model and the MWC Model

Both our new model, which is closely linked to the Adair-Klotz model [6,7], and the MWC model can be used equally well to quantify the Ca^2+^-binding kinetics of CR. However, we consider the new model to facilitate the “intuitive” understanding of how the kinetic properties of the cooperative sites relate to the binding kinetics at the level of the whole protein or at the macroscopic level. The Adair-Klotz model is the most general description of equilibria in terms of stochiometric binding. It describes the steady-state equilibrium using the constants (*K*_1_, *K*_2_….*K*_n_) for the successive binding (or macroscopic) steps, but not as the affinity constants of the individual (or microscopic) binding sites:

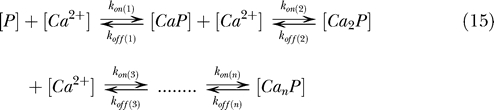
where

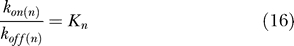
for which in equilibrium, the fractional occupation (*ν*) of a protein *P* is described by the Adair-Klotz equation:

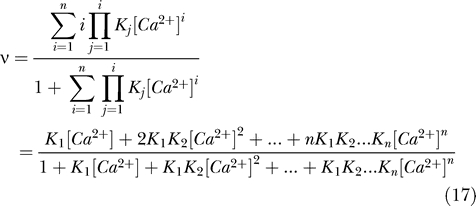



Usually, rate constants are not denoted in the macroscopic equilibrium equation (Equation 15); instead, only the *K* values are denoted, which is sufficient for steady-state descriptions. The equilibrium constants for the new model (*K*_T_ and *K*_R_) and the MWC model (*K*_T_, *K*_R_, and *L*_0_) can often rather easily be translated into macroscopic *K* values [10] (also see Protocol S1). Therefore, it is fairly simple to relate any of the steady-state constants of cooperative models (new, MWC, and KNF) to the more generally used Adair-Klotz equation.

In addition to the calculation of the steady-state equilibrium (Equation 17), the macroscopic Adair-Klotz model compiles the binding of multiple binding sites into an intuitively easy-to-understand sequential binding model. It would even be more insightful if one could also obtain the rate constants for each macroscopic step. Unfortunately, the macroscopic rate constants are generally extremely hard to define when cooperative mechanisms are involved. For example, the macroscopic *k*_on(*1*)_ for the MCW model depends on the relative amounts of totally unoccupied molecules in the R and T states (see Figure 2B). At steady state, this equilibrium is fairly straightforward, as this is simply defined by *L*_0_. However, when the balance is disturbed by a sudden change in Ca^2+^ concentration, it will disturb the equilibrium between unoccupied molecules in the R and T states. This equilibrium will settle over some time according to *k*_+_ and *k*_−_. During this time, the relative amount of binding sites in states R and T is dynamically changing, making the macroscopic *k*_on(*1*)_ itself dynamic. With most cooperative models, the macroscopic rate constants will be dynamic because they are dependent on most perturbations. This makes the rate constants very difficult to interpret (and to calculate).

However, for the new model of cooperativity, the macroscopic rate constants are easily defined and are truly constant. For instance, for two cooperative sites as described in this paper:

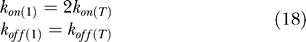
and

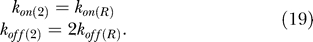



Through these simple relationships and according to our data, CR can be quantitatively described as a mixture of two “virtual” CaBPs. The cooperative part can be described as:

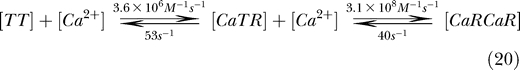
and the independent part as:

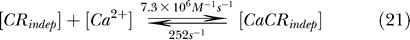
where

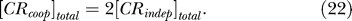



### New Insights into the Regulation of Cellular Ca^2+^ by CR

**Figure 7:**
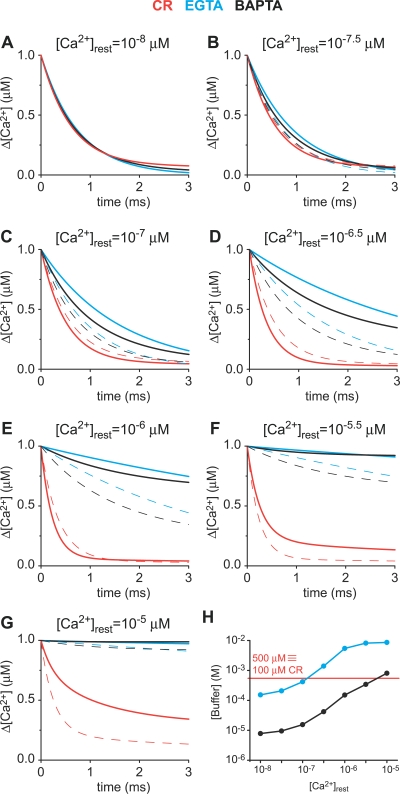
Kinetic Behavior of CR Determined with the Nonlinear Kinetic Parameters The graphs represent simulated Ca^2+^ signals induced by 1 μM steps in [Ca^2+^] in the presence of 100 μM CR (red lines), 153 μM EGTA (blue lines), or 7.8 μM BAPTA (black lines). (A) The simulated Ca^2+^ signal induced by a 1 μM step in [Ca^2+^] from a starting [Ca^2+^] of 10 nM in the presence of 100 μM CR resulted in the red [Ca^2+^] decay curve. This trace was used to model the concentrations of EGTA or BAPTA needed to best fit the CR [Ca^2+^] decay curve, and were found to be either 153 μM (EGTA; blue trace) or 7.8 μM (BAPTA; black trace). (B–G) The same step in [Ca^2+^] of 1 μM was then “induced” from increasing resting [Ca^2+^](solid lines). The [Ca^2+^] decay curves from the previous image (lower resting [Ca^2+^]) were depicted as dashed lines (e.g., the dashed lines in [C] are the solid lines from [B]) to highlight the “shift” of the curves as the resting [Ca^2+^] increases. If the solid line is below the dashed line of the same color, the [Ca^2+^] decay becomes faster, and vice versa, a solid line above the dashed line indicates a slowing of the [Ca^2+^] decay. The decay in the presence of CR (red) becomes faster when the starting [Ca^2+^] increases from 10 nM to 1 μM (A–E) and then slows down from 1 μM to 10 μM. (H) shows the concentration of either EGTA (blue) or BAPTA (black) needed to mimic the buffering of a 1 μM step in [Ca^2+^] by 100 μM CR at different resting [Ca^2+^]. The intercept between the red line with the blue and black curves indicate at what starting [Ca^2+^] the synthetic buffers closely mimic a 1 μM step in [Ca^2+^] in the presence of 100 μM CR. Note that since CR has five Ca^2+^-binding sites and EGTA and BAPTA only one each, 100 μM CR was considered to be equivalent to 500 μM EGTA or BAPTA. Thus, for the selected 1 μM step in [Ca^2+^] at a lower starting [Ca^2+^] (approximately 100 nM), CR apparently behaves more like EGTA, whereas at higher starting [Ca^2+^] (>3 μM), CR works more like BAPTA.

As described above, at a starting [Ca^2+^] around the apparent *K*_d_ for the cooperative binding sites, CR will more effectively buffer perturbations at lower frequencies (Figure 6). Thus, the Ca^2+^-buffering kinetics of CR clearly depends on the starting [Ca^2+^] that determines the distribution between states T and R of the cooperative binding sites. While more cooperative sites get occupied, more Ca^2+^ binding will take place through the second faster binding step as described in Equation 20. To better understand the cooperative nature of Ca^2+^ binding by CR, we simulated with the new model a 1 μM step in [Ca^2+^] from a resting [Ca^2+^] of 10 nM in the presence of 100 μM CR. In comparison, we also simulated the widely used synthetic Ca^2+^ buffers BAPTA (*K*_d_ = 160 nM, *k*_on_ = 2 × 10^8^ M^−1^s^−1^, Maxchelator software version 10/02, see Materials and Methods) and EGTA (*K*_d_ = 70 nM, *k*_on_ = 1 × 10^7^ M^−1^s^−1^ [2]). Under these conditions, the [Ca^2+^] decay kinetics mediated by 100 μM CR could be faithfully reproduced by either 7.8 μM BAPTA or 153 μM EGTA (Figure 7A). Now, to compare the binding kinetics of CR to the two synthetic chelators without cooperative binding, we kept all parameters constant, i.e., 1 μM steps in [Ca^2+^], buffer concentrations of CR, BAPTA, and EGTA, and only varied the resting [Ca^2+^] (10 nM to 10 μM) from which the [Ca^2+^] step was induced. As the resting [Ca^2+^] increases, the [Ca^2+^] decay kinetics in the presence of either one of the noncooperative chelators BAPTA and EGTA is slowed down (Figure 7A–7G). This slowing results from the fact that at higher initial [Ca^2+^], more BAPTA or EGTA molecules will be in the Ca^2+^-bound form, and thus, less Ca^2+^-free buffer is available. In contrast, as the initial [Ca^2+^] increases from 10 nM to approximately 1.1 μM, CR speeds up the decay in [Ca^2+^] and buffers Ca^2+^ faster until approximately 1.1 μM resting [Ca^2+^], above which CR behaves “similarly” to EGTA or BAPTA. More simulations have indicated that the exact breaking point between this novel behavior and classic behavior is dependent on the CR concentration and the Ca^2+^ step size; however, the breaking point is always close to 1.1 μM for CR. Evidently neither EGTA nor BAPTA are able to mimic the properties of CR over the whole physiological range of [Ca^2+^]. Yet for a given resting [Ca^2+^] and a defined step increase in [Ca^2+^], a BAPTA or EGTA concentration can be found that will closely mimic the effect of CR on the [Ca^2+^] decay kinetics. But with only a slight change in the initial [Ca^2+^] or the step size, this particular concentration of the synthetic chelator will not accurately reflect the action of CR. As an example, the concentrations of BAPTA or EGTA needed to mimic Ca^2+^ binding by CR for the simulations (Figure 7A–7G) are shown in Figure 7H. Since CR has five binding sites, 100 μM CR is, in terms of Ca^2+^-binding sites, equivalent to 500 μM of either EGTA or BAPTA. For the example shown, at initial [Ca^2+^] smaller than 0.3 μM, the concentration of EGTA needed to mimic 100 μM CR is in that same order of magnitude (∼150–1,400 μM). The amount of BAPTA necessary to mimic CR is on the order of 500 μM when the initial [Ca^2+^] is approximately 1–10 μM (∼150–800 μM, respectively) (Figure 7H). Thus, one can conclude that CR behaves EGTA-like around physiological resting [Ca^2+^] (20–100 nM) typically seen in neurons and more BAPTA-like at the higher [Ca^2+^] observed during bouts of activity.

## Discussion

### Quantifying Kinetics of Cooperative Binding

The kinetic properties of Ca^2+^ binding to CR were quantified using two different models featuring cooperativity between Ca^2+^-binding sites. The quantification of ligand binding to a protein with multiple binding sites is inherently difficult. First of all, when assuming that all binding sites are never truly identical, then for each binding site, at least two constants need to be determined. When these binding sites cannot be studied individually, the number of variables to be simultaneously determined quickly increases in a mathematical description of the protein. Consequently, degrees of freedom in the model will increase, which will decrease the accuracy and enhance the variability of the fitting. These difficulties are further exacerbated by cooperative binding, in which properties of the binding sites dynamically change, resulting in even more variables to determine. Although it is unlikely that any two binding sites are truly identical, a general way to decrease the number of variables is to assume the different binding sites to be identical in binding and allosteric behavior [12,13]. Even with such simplifications, the number of variables remains fairly large. To overcome this problem, we performed our experiments at a variety of initial conditions and at a variety of uncaging energies. In this way, we created a large set of data that varied by known parameters. We used a bootstrap method to create several datasets to be fitted simultaneously. The fit routine was set up in such a way that the models were required to consistently describe the properties of CR simultaneously over the whole range of variations in experimental conditions within one set, as well as over the whole range of uncaging energies. This technique resulted in (log)-normally distributed results so that erroneous fit results could be easily identified among the fits to any individual set.

Initial studies on CR have revealed the protein to contain cooperative Ca^2+^ binding sites as evidenced by Hill coefficients of 1.3 or 2.4 [4] and 1.9 [22] for human and chick CR, respectively. Thus, this CaBP appeared to be well suited to serve as a model to quantitatively describe its cooperative Ca^2+^-binding properties. Even if such a description would fail to unravel the “exact” physical interpretation of Ca^2+^ binding to CR, the obtained data were expected to serve as a powerful tool to understand the role of CR in shaping intracellular Ca^2+^ dynamics in neurons. To quantify the cooperative binding to CR, two models were tested: a newly developed one and the MWC model. The latter was chosen because it has been used earlier to determine cooperative binding to biologically active molecules [14,16]. These studies were limited to special cases where transition rates between allosteric isoforms are much slower than the binding rates [14,15] or where binding and unbinding rates could be measured independently [16]. Here, we showed that it is feasible to determine the rate constants of CR using a MWC model outside of these constraints. In addition, we developed a different model closely related to the Adair-Klotz model [6,7], in which we did not account for the transition rate between the two possible states for a cooperative binding site. Both models yielded a similar quantitative description of CR's cooperative properties, but as long as crystal structures of CR in different states of Ca^2+^ occupancy remain unknown, the exact physical interpretations of Ca^2+^ binding to CR will not be available.

### Properties of Cooperativity

Within one molecule, cooperativity can only be established by sets of at least two binding sites that change their properties based on the occupancy by Ca^2+^. In both models used here, four cooperative binding sites can occur in either the T state with low affinity for Ca^2+^ or R state with high affinity for Ca^2+^. The dualistic nature of the binding sites causes the classical cooperativity as seen in steady-state binding studies in which the four cooperative sites in CR can be described with a *K*_d_ of 2.5 μM and a Hill coefficient of approximately 2 [4,22]. The steady state properties for the cooperative sites of either the new model (Figure 5A and Table 1, *K*_d(app)_ = 1.4 μM; and Figure 5E and Table 1, *n*_H_ = 1.9) or the MWC model (Figure 5A and Table 2, *K*_d(app)_ = 1.5 μM; and Figure 5E and Table 2, *n*_H_ = 1.9) are in close agreement with the steady-state values for CR. Also for the independent site, the *K*_d_'s found with the new model (Figure 4A and Table 1, 36 μM) or MWC model (Figure 4A and Table 2, 41 μM) are in agreement with the earlier determined value (53 μM) by steady-state measurement [4]. The mechanism to create positive cooperativity with an initial low-affinity binding step as expressed in Equation 20 for the new model can be quite confusing. Since the first Ca^2+^-binding step is with a binding site in the low-affinity T state, it may appear that this will be limiting for the whole process, so that overall binding will only occur at higher [Ca^2+^]. However, it should be considered that even at lower [Ca^2+^] (e.g., at 100 nM inside a cell), at equilibrium. some of the sites in the T state will bind Ca^2+^, yielding some cooperative pairs with one Ca^2+^ bound, which then rapidly leads to some fully occupied cooperative pairs:

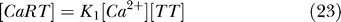


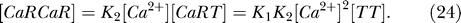



Since the second step in this process is governed by a high-affinity site, a considerable number of the cooperative pairs will be fully occupied. The combination of these reaction steps gives rise to the intermediate apparent *K*_d_ (*K*_d(app)_) (also see Equation 6 and, in Protocol S1, Equation S76). Although high-affinity R sites (short for “a site in the R state”) only become available after Ca^2+^ binding to CR's low-affinity T sites, they still play a determining role for the steady-state equilibrium. In dynamic situations, the slower binding of Ca^2+^ to a T site has to precede binding of a second Ca^2+^ to a faster R site, the former step apparently being rate limiting. However, at a given initial [Ca^2+^], CR molecules are present in different states (metal-free, T state, R state, and completely Ca^2+^-bound) according to the parameters described in Tables 1 and 2; one example is described in more detail here. Assuming a 10 μM step in [Ca^2+^] from a resting [Ca^2+^] of 100 nM in the presence of 100 μM CR, the initial ratio for [unoccupied T sites]/[unoccupied R sites] is 395 μM/1.4 μM (calculated with Equations S34–S36 in Protocol S1). Of the 10 μM increase in Ca^2+^, almost all will be buffered by CR: 5.6 μM will bind to T sites, 3.9 μM will bind to R sites, and 0.35 μM to the independent site V. Evidently, the initially available R sites (1.4 μM) will not be sufficient, and most of the bindings to the R site will be time-limited by initial binding to T sites. However, at a resting [Ca^2+^] of 1 μM, the initial ratio of unoccupied T*/*R sites is 250 μM/9 μM. In this case, 5 μM Ca^2+^ will bind to T sites, 4.8 μM will bind to R sites, and 0.15 μM to site V. Thus, enough sites (9 μM) in the fast R state will be available and allow for fast buffering, not necessitating the slower stepwise change from the T to the R state. Model simulations of these experiments indicate that the surplus of sites in the R state will, within approximately 1 ms, bind up to 0.7 μM Ca^2+^ more, which at a later time shifts to a CR molecule in the T state. By acting as a temporary substitute, the free sites in the R state can even “speed up” the eventual buffering of binding to sites in the T state. Albeit the initial amount of free sites in the R state is relatively small, it will significantly contribute to the buffering speed at initial [Ca^2+^] around *K*_d(app)_. Also, the low-affinity independent site, which will hardly play a role in the steady-state buffering, initially binds up to 1.8 μM Ca^2+^ which is later “transferred” to sites in the T state and will contribute to the overall buffering speed. However, this contribution is virtually identical when starting from either 100 nM or 1 μM Ca^2+^.

### Unusual Nonlinear Properties of CR

To circumvent the difficulties of “real” CaBPs encountered in electrophysiological experiments (e.g., washout, unknown concentrations, and kinetic properties), they are often substituted by the artificial Ca^2+^ buffers BAPTA and EGTA. Since BAPTA and EGTA have significantly different on-rates (2 × 10^8^ M^−1^s^−1^ and 1 × 10^7^ M^−1^s^−1^, respectively), it is generally assumed that a process influenced by BAPTA, but not by EGTA, must have Ca^2+^-binding on-rates comparable to ones of BAPTA. So far, two studies [28,29] have inferred the kinetics of Ca^2+^ binding by CR from comparing it to BAPTA and EGTA.

It was thus surmised that CR must have one or several binding sites with fast on-rate(s) [28]. The conclusion was drawn from the finding that addition of BAPTA, but not EGTA, to CR-deficient cells could rescue the CR deficiency. However, such generalizations are certainly error-prone, because both EGTA and BAPTA have *K*_d_ values much lower than CR. This will considerably affect the results, since the speed of buffering is also dependent on the concentration of free sites and not only on the rate constants. Under resting conditions inside cells (∼100 nM [Ca^2+^]), at least 40% of either EGTA or BAPTA are occupied by Ca^2+^ ions and do not add to the buffering speed. Our findings show that if BAPTA (or EGTA) can replicate a cellular buffering process under certain experimental conditions defined by, e.g., initial resting [Ca^2+^], step size, and geometry of Ca^2+^ influx, it does necessarily mean that the cellular buffering is “comparable to BAPTA or EGTA” and therefore “fast” or “slow,” respectively.

The exact intracellular distribution of CR is not well understood [30]; although principally considered as a cytosolic protein, a fraction of CR molecules could be anchored to specific sites [31–33], leading to higher local concentrations, possibly at Ca^2+^ hotspots, as suggested for calmodulin around the L-type Ca^2+^ channel [34]. Such local accumulation could lead to local high buffering speeds, especially at hotspots, where the higher local Ca^2+^ concentrations would drive CR into a faster mode. Consequently, if CR is concentrated at certain subcellular compartments, the concentration of freely diffusing CR molecules will be lower. And if this freely moving CR is only confronted with smaller Ca^2+^ signals, this will result in slower CR Ca^2+^-buffering kinetics. Slower properties of CR at lower [Ca^2+^] together with its suggested mobility (a diffusion coefficient of approximately 20 μm^2^s^−1^, assuming a similar mobility as the closely related CaBP CB [29,35]), supports the notion that part of CR will slowly bind and release Ca^2+^ and thus act like a “slow” buffer. In contrast, the faster Ca^2+^ buffering of CR at Ca^2+^ hotspots or when present in the “fast” mode, i.e., with one of the cooperative sites in the Ca^2+^-bound form, may explain earlier findings that BAPTA could functionally rescue CR deficiency [28,36]. According to our findings, “simple” Ca^2+^ chelators (EGTA and BAPTA) can never fully replicate certain functional aspects of CaBPs, because the complexity of Ca^2+^ binding to “real” CaBPs such as CR cannot be mimicked by small synthetic Ca^2+^ buffers lacking cooperativity.

The steady-state aspect of cooperative binding has been reported and analyzed in detail for Ca^2+^ sensors such as calmodulin (for a review, see [3]). Cooperativity was also reported for CB [37,38], a protein with sensor and buffer functions [35,38], but the quantitative aspects of cooperativity have not yet been investigated in detailed steady-state binding studies. Nevertheless, cooperative binding has not been modeled in a CaBP to examine its effect on cellular Ca^2+^ transients. Our results on CR pave the way to more realistically model intracellular Ca^2+^ dynamics, thus leading to a better understanding of the spatial and temporal actions of Ca^2+^ within a cell. The importance of correctly determining the physiological actions of CaBPs was recently shown in *Xenopus* oocytes. The effect of parvalbumin (PV), a CaBP with two Ca^2+^ binding sites and minimal cooperativity [39], could be closely mimicked by the synthetic slow Ca^2+^-buffer EGTA [29]. Yet, the effect of CR on IP_3_-mediated Ca^2+^ release was significantly different from that of the fast Ca^2+^-buffer BAPTA, in particular at low [IP_3_], when Ca^2+^ elevations were small. Under these conditions, CR caused a leftward shift in the concentration-response relationship as observed with the slow buffer PV. Under the same conditions, CR produced localized Ca^2+^ transients or “puffs,” a phenomenon never observed in the presence of BAPTA [28]. Our findings strongly support the hypothesis [29] that the kinetic properties of individual CaBPs are finely tuned to specific cellular functions and may explain the need for a large number of CaBPs (more than 240 EF-hand–containing proteins) detected in the human genome [40]. Our novel approach determining the cooperative kinetics of Ca^2+^ binding to CaBPs will lead to a better understanding of their highly specialized roles in cellular Ca^2+^ signaling. In general, the method should also be applicable to any CaBP or to other multisite cooperative binding processes. This is expected to yield a more detailed understanding how CaPBs shape the spatiotemporal aspects of Ca^2+^ signaling. Our findings may also help to reconcile some reported discrepancies concerning CR's function and putative effects on biological processes. The new model devised in the present study extends the analysis of cooperativity beyond the static steady-state condition, providing a powerful tool for the investigation of the dynamics and functional significance of cooperative binding in general.

## Materials and Methods

### Solutions.

All experiments were performed in solutions containing 120 mM KCl, 40 mM HEPES (pH set at 7.30), 100 μM Oregon Green BAPTA-5N (OGB-5N; Molecular Probes), a varying amount (4–17 mM) of DM-nitrophen (DMn, (4,5-dimethoxi-2-nitrophenyl)-1,2-diaminoethane-N,N,N′,N′-tetrasodium salt; Calbiochem) and CaCl_2_. In selected experiments, the solution also contained calretinin (CR). Because DMn is extremely sensitive to light and might uncage spontaneously, solutions were freshly prepared before every experiment. The light source in the experimentation room was equipped with a yellow filter (500 nm long-pass) to avoid unwanted photolysis. The concentration of fresh stock solutions of DMn (15 or 30 mM) was determined photometrically at 350 nm (Hewlett-Packard 8453) using an extinction coefficient of 4.33 × 10^3^ M^−1^cm^−1^ for DMn [26]. For these measurements, small samples of the final experimental solutions were diluted to yield [DMn] of approximately 50 μM in a solution containing 4 mM EGTA to remove any free Ca^2+^. Most stock solutions contained approximately 80%–90% of the expected DMn concentration based on the manufacturer's specifications. The basis of the discrepancies between the expected and measured DMn concentrations is difficult to determine and might result from impurities; it is conceivable that some of the compounds decayed due to inadvertent illumination. To accurately account for the amounts of Ca^2+^ cage, the actual [DMn] in our experimental solutions were calculated, and the corrected values were used in our analyses. Experiments were carried out at room temperature (∼25 °C). All chemicals were obtained from Sigma-Aldrich, unless otherwise mentioned. Values are expressed as mean ± the standard error of the mean.

### Solution preparation and steady-state [Ca^2+^] measurements with Ca^2+^-sensitive electrodes.

The initial free [Ca^2+^] of each solution was titrated to be between around 2 μM or 8 μM (indicated in Results), using custom-made Ca^2+^-selective electrodes [25]. As opposed to earlier experiments [25] in which the *K*_d_ of the Ca^2+^ buffer in standard solutions was regulated by changing the pH [41], here we made KCl-based pCa (−log[Ca^2+^]) standard solutions with an ionic strength of 120 mM and pH around 7.3 (see Protocol S1, Table 1) using Maxchelator software version 10/02 (http://www.stanford.edu/~cpatton/maxc.html). These standard solutions were used for calibrations; the standard pCa 6 solution was used as a reference solution in the Ca^2+^ electrodes.

To obtain the experimental solutions, Ca^2+^-free solutions containing DMn, OGB-5N, and CR (in the case of control solution without CR) were titrated with aliquots (1–3 μl) of the same solution containing 1–100 mM CaCl_2_. After thoroughly mixing every added aliquot, the [Ca^2+^] was measured with the Ca^2+^-sensitive electrodes. This procedure was repeated until the sought concentration was reached. All [Ca^2+^] were verified in the uncaging experiments by comparing baseline fluorescence (*F*_rest_) with the maximal possible fluorescence (*F*_max_) for that solution. *F*_max_ was determined by repetitive uncaging of Ca^2+^ in some of the samples until fluorescence did not increase any further (so that [Ca^2+^] ≫*K*_d(OGB-5N)_). The [Ca^2+^] was calculated using the following equilibrium formula:

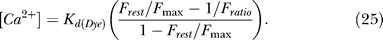



### Dynamic Ca^+^ measurements.

To measure the dynamics of Ca^2+^ binding to CR with high temporal resolution, we used UV-flash photolysis of DMn. We used a setup that was described earlier [2,25,42,43]. Briefly, it consisted of a small chamber (20 μl) mounted on an inverted microscope equipped for epifluorescence (IM35; Carl Zeiss) with a 505-nm dichroic mirror and 510 LP emission filter (Chroma Technology). In the chamber, the polished end of a silica multimode optical fiber (Ø 800 μm; Thorlabs) was mounted to deliver 20-ns flashes of UV light (347 nm) from a frequency-doubled ruby laser (Lumonics) to photolyze DMn. To excite the OGB-5N molecules, an argon laser (488 nm, 1 W; model 95; Lexel) was focused through the epifluorescence illumination port of the microscope with a 20× objective (Fluo20; Nikon), forming a small illumination spot directly in front of the optical fiber. The relatively small spot size of the excitation light (1–10 μm) compared to the large area of UV illumination (cone with minimal of Ø 800 μm) ensured minimal diffusion effects during the time span (200 ms) of [Ca^2+^] changes. The [Ca^2+^] transients changed substantially only when the illumination spot was moved towards the outer edge of the optical fiber, i.e., to the edge where uncaging took place. This indicated that diffusion artifacts between areas of UV illumination (uncaging) and no UV illumination (no uncaging) on the timescale of our measurements occur only very close to the edge of the UV-illuminated area and not in the area where data were collected. The fluorescence of OGB-5N was measured with a photodiode (PIN-HR008; UDT Sensors) in the focal plane of the microscope. The small diameter of the photodiode (200 μm) minimized errors caused by diffusion in the *z*-axis. Despite using appropriate optical filters and excitation spectrum of OGB-5N peaks at 494 nm, the high-energy UV flashes still induced brief, but large, optical transients that saturated the detection system. To avoid these artifacts, a patch clamp amplifier (Axopatch 200A; Axon Instruments) with an integrating headstage was used to measure the currents generated by the photodiode. The feedback capacitor of the headstage was short-circuited (reset), and its readout was blanked exactly at the time of the UV flash so that there was no signal measured at the instant of the flash. The analog signal was low-pass filtered with the eight-pole low-pass Bessel filter of the amplifier at 10 kHz, digitized at 50 kHz (PCIO-MIO-16XE-10; National Instruments), and sampled on a PC with a custom-made program (EVAN) written in LabView (National Instruments). A pulse generator (4000 PG; Neuro-Data Instruments) was used to trigger the sampling, UV laser, headstage reset and blanking, and the shutter for the OGB-5N excitation light.

For a typical experiment, an approximately10-μl droplet of DMn solution was placed in the recording chamber. During each flash, only 0.05% to 1.5% of the DMn in the spot directly in front of the optical fiber was uncaged (predicted by our model; see Results). However, to avoid significant changes in baseline conditions due to excessive uncaging of DMn or evaporation, no more than three flash-evoked transients were acquired from each droplet. Measurements in the same droplet were performed at least 1 min apart to ensure that all the components in the droplet returned to steady-state baseline conditions. The data were stored for offline analysis of the fluorescence transients with a computer model (see below).

### Properties of OGB-5N.

We used the low-affinity dye OGB-5N because of its fast kinetics of Ca^2+^ binding and unbinding needed for tracking the expected rapid changes in [Ca^2+^]. The properties of the dye were determined as previously described [25]. For one batch of the dye (lot# 34B1–2; Molecular Probes ), we found a *K*_d_ of 29.3 μM, a *k*_off_ of 7.52 × 10^3^ s^−1^, a *k*_on_ of 2.6 × 10^8^ M^−1^s^−1^, and an *F*_min_/*F*_max_ ratio of 10.8. For another used batch (lot# 15C1–2), we measured a *K*_d_ of 36.1 μM, a *k*_off_ of 8.67 × 10^3^ s^−1^, a *k*_on_ of 2.4 × 10^8^ M^−1^s^−1^, and an *F*_min_/*F*_max_ ratio of 40.0. These values were used in the mathematical model (see below) to describe the properties of the two batches of OGB-5N used in the various experiments. We have no explanation for the variability between these two batches other than the fact that specific contaminations might occur in different batches from the supplier (Molecular Probes, personal communication).

### Properties of DMn.

For each group of experiments, we determined the properties of DMn independently by uncaging experiments with no protein present, as described before [25]. These properties of DMn were then set for that specific experiment to compensate for possible differences between DMn batches. The observed properties of DMn were comparable to ones previously found (see Table 1 in Protocol S1) [25].

### CR purification and determination of CR concentrations.

Human recombinant CR was expressed in Escherichia coli and purified with a series of chromatographic steps as described before [4,44]. The purity of the isolated protein was estimated to be greater than 98% as judged from bands on SDS polyacrylamide gels (unpublished data). The initial protein concentration was determined by absorption measurements at 280 nm and using a molar extinction coefficient ɛ_280nm_ of 26,860. Small aliquots of the protein (100–500 μg) in 10 mM (NH_4_)HCO_3_, 0.1 mM CaCl were lyophilized and then reconstituted directly in the solutions used for the uncaging experiments. To accurately determine the protein concentrations of all solutions used for the uncaging experiments, 10–15-μl samples were removed, stored at −20 °C, and simultaneously measured at the end of the series. The protein concentration was measured using a detergent-compatible assay based on a folin-phenol reagent (Bio-Rad) and using bovine serum albumin (BSA) as standard. All samples were measured in duplicates. Initial tests with solutions containing DMn and OGB-5N revealed that the colorimetric effect of these compounds was negligible at the concentrations present in the experimental solutions. The accuracy of the concentration measurements was validated by one round of fitting, in which the CR concentrations were fitted by the model, while the kinetic rates were allowed to deviate maximally 10% from their expected value. These fits confirmed the results of the protein assay.

### Data analysis.

All data were analyzed using MS Excel (Microsoft) and Berkeley Madonna 8.0 (University of California Berkeley). To determine the kinetic parameters (association and dissociation rates) from the fluorescence recordings, we used a mathematical model build in the ordinary differential equation solver Berkeley Madonna 8.0 that incorporates all of the reactions in the uncaging solution (Figure 1). The DMn uncaging and OGB-5N signaling part of this model was used earlier to determine the exact properties of DMn [25]. This model was expanded with a part to simulate the binding of Ca^2+^ to CR (see Figure 1B, and for a complete description of the models, see Protocol S1).

The model fit the simulations to the fluorescence recordings by iterating the parameters with the fourth-order Runge-Kutta method. The output of the model is:

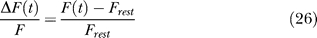
where *F*(*t*) is the fluorescence acquired over time (*t*) with *t* = 0 at the time of the flash, and *F*_rest_ is the resting fluorescence averaged over 50 ms before flash delivery_._ To more accurately fit the fast-rising phase while avoiding bias from late slow-decaying phase of the fluorescence transients, data points were omitted exponentially towards the end of every trace the fitting routine.

## Supporting Information

Figure S1 Ca^2+^ Transients Grouped According to the Experimental Conditions All individual Ca^2+^ transients are grouped according to the experimental conditions as mentioned in the table. The red traces are the traces shown in Figure 3 of the paper. In the table for the OGB-5N column, I refers to lot number 34B1–2 and II to lot 15C1–2.
(594 KB PDF)

Figure S2 Pseudo-Random Picking of Fit Sets Flowchart of the compilation of 38 randomly selected sets of 14 traces derived from seven groups of data shown in Figure 3 of the paper. Random sets of traces were picked from the seven groups of data; from every group, two traces were picked per set. For the 38 sets, 76 picks are needed from every group. This means that every trace has to be picked 76/*n* times (*n*'s are not equal for each group, see table), if every trace of a group is to be picked an equal number of times. Since 76/*n* is most likely not an integer, we picked every trace at least *X* times, where *X* is the closest smaller integer than 76/*n* (see table). To reach the number of 76 picks, 76 − *n* × *X* = *Y*, see table) traces have to be picked one more time (*X* + 1 times in total). By picking the traces in the way described here, we ensure that each trace within a group is used at approximately the same frequency for the fit sets.
(210 KB PDF)

Figure S3 Properties of the Independent Site and the Four Cooperative Sites Fit with *n*_H_ = 1 The figures shown here are identical to Figure 4 (here [A] and [B]) and Figure 5 (here [C] and [D]) in the paper. Depicted here are the fit results for fitting with the new model in red (and pink for apparent *K*_d_ in [C]). The results for the MWC model (blue symbols in the paper) are omitted for clarity. The black symbols are the fit results when a model for CR was used in which the four cooperative sites are simulated when *n*H = 1 (no cooperativity). Although fitting the uncaging curves with such a model gives reasonable fits, the results (black symbols) of most of the fitted parameters showed strong deviations when using *n*_H_ = 1 for the four cooperative sites. This indicated that there is no unique solution to describe CR's Ca^2+^-binding properties without cooperativity, in line with previous steady-state findings of *n*_H_ values.
(298 KB PDF)

Protocol S1 Supplemental Methods Consisting of Four Sections (1) Supplemental Table 1: Properties of DMn determined from the seven groups of experiments.
(2) Supplemental Table 2: KCl-based pCa standard solutions used to calibrate Ca^2+^-sensistive electrodes and to determine the *K*_d_'s of various OGB-5N lots.
(3) Complete description of the mathematical model (the Berkeley Madonna model) describing the reactions occurring in the experiments represented in a schematic and a mathematical way.
(4) Description of the mathematical relationship between the Hill equation and the new and the MWC models via the Adair-Klotz equation for two binding sites.
(624 KB DOC)

## Abbreviations

CaBP: Ca^2+^-binding protein
CB: calbindin
CR: calretinin
DMn: DM-nitrophen
MWC: Monod-Wyman-Changeux
OGB-5N: Oregon Green BAPTA-5N
