# Supplementary figures and images for: Resolving the Fast Kinetics of Cooperative Binding: Ca2+ Buffering by Calretinin
Source: PLoS Biol. 2007 Nov 27;5(11):e311. doi: 10.1371/journal.pbio.0050311 (PMC2229850; doi:10.1371/journal.pbio.0050311)

| figure   | <i>n</i> | $[Ca^{2+}]_{free} (\mu M)$ | $[CR]_T (\mu M)$ | $[DMn]_T (mM)$ | OGB-5N |
|----------|----------|----------------------------|------------------|----------------|--------|
| <b>A</b> | 12       | 2.1                        | 20               | 4.7            | I      |
| <b>B</b> | 13       | 2.1                        | 40               | 4.7            | I      |
| <b>C</b> | 21       | 2.4                        | 31               | 4.4            | I      |
| <b>D</b> | 21       | 2.4                        | 62               | 4.4            | I      |
| <b>E</b> | 21       | 5.3                        | 135              | 18.0           | II     |
| <b>F</b> | 10       | 4.9                        | 85               | 16.3           | II     |
| <b>G</b> | 25       | 2.0                        | 390              | 14.6           | II     |

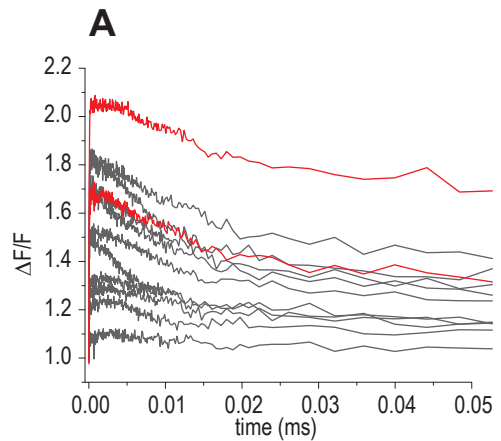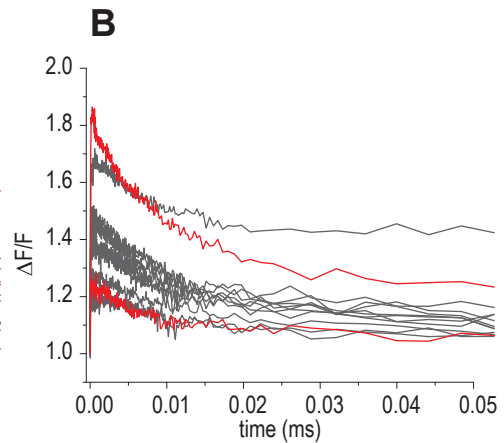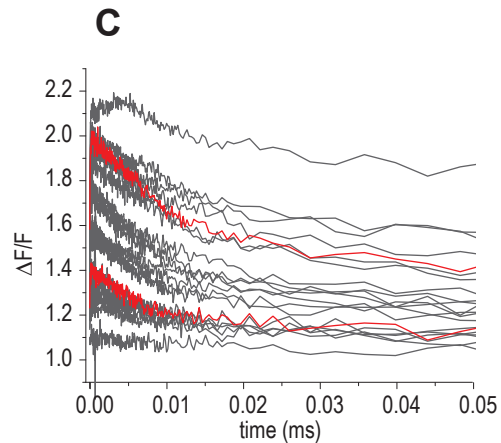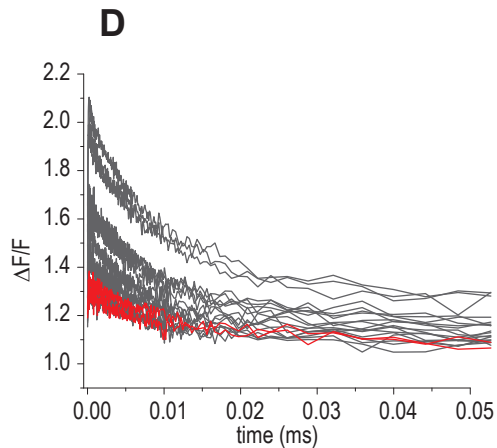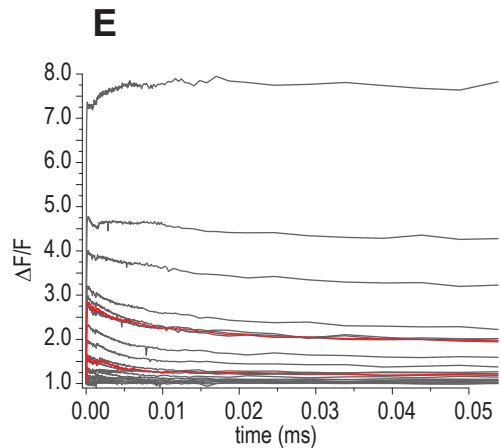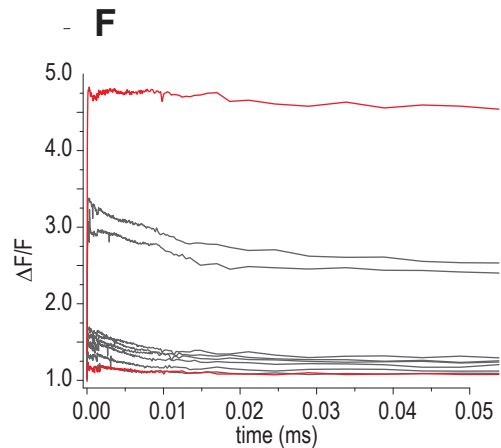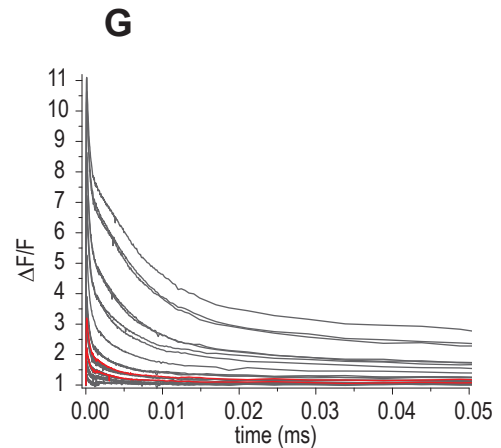

Supplement: Figure S1 — All individual Ca2+ transients are grouped according to the experimental conditions as mentioned in the table. The red traces are the traces shown in Figure 3 of the paper. In the table for the OGB-5N column, I refers to lot number 34B1–2 and II to lot 15C1–2. (594 KB PDF) [file pbio.0050311.sg001.pdf]
