# Supplementary material for: Resolving the Fast Kinetics of Cooperative Binding: Ca2+ Buffering by Calretinin
Source: PLoS Biol. 2007 Nov 27;5(11):e311. doi: 10.1371/journal.pbio.0050311 (PMC2229850; doi:10.1371/journal.pbio.0050311)

group	number of traces (n)	min. number of picks per trace needed (X)	number X+1 picks needed for this group (Y)
I	12	6	4
II	13	5	11
III	21	3	13
IV	21	3	13
V	21	3	13
VI	10	7	6
VII	25	3	1

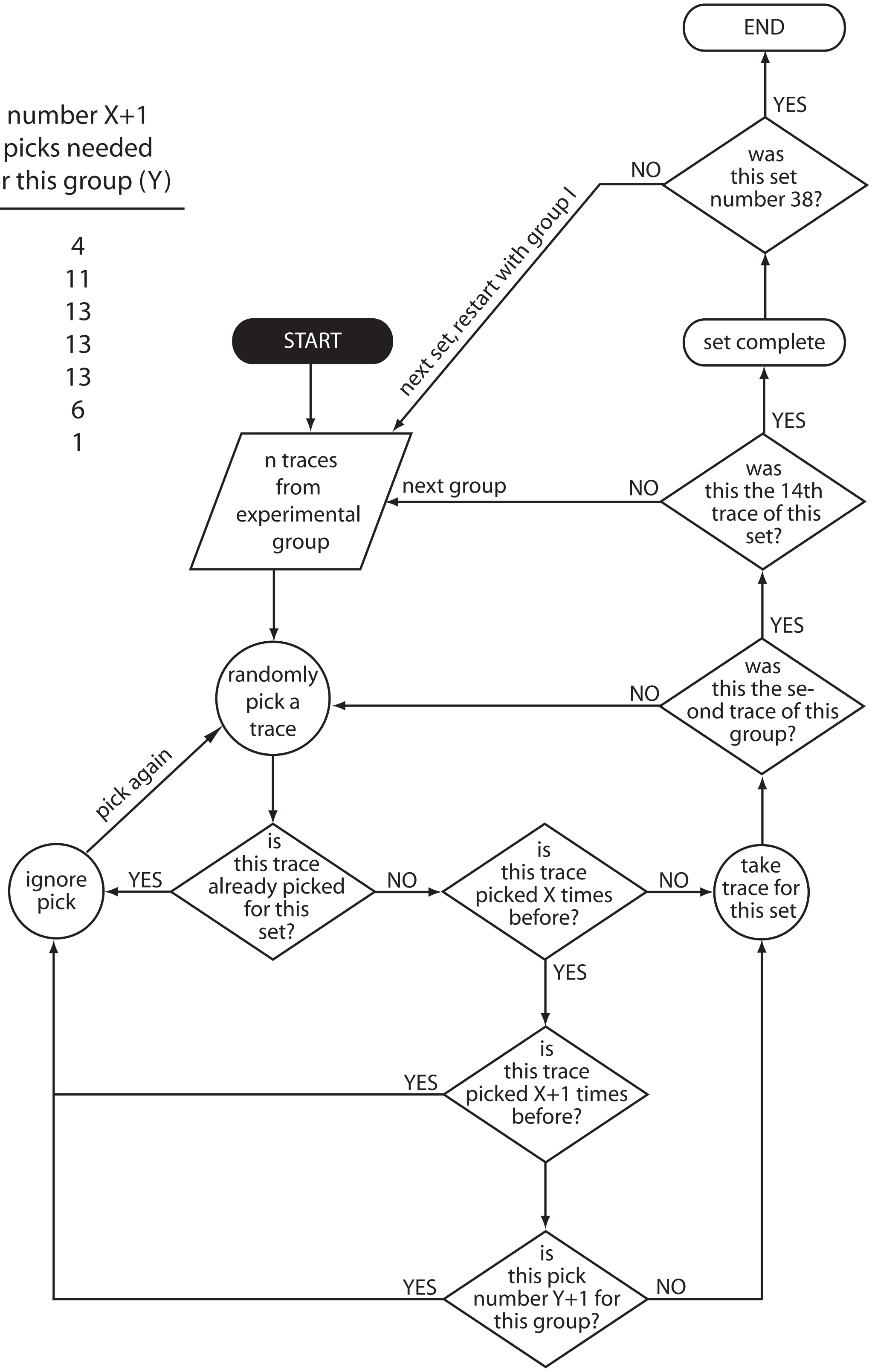

Supplement: Figure S2 — Flowchart of the compilation of 38 randomly selected sets of 14 traces derived from seven groups of data shown in Figure 3 of the paper. Random sets of traces were picked from the seven groups of data; from every group, two traces were picked per set. For the 38 sets, 76 picks are needed from every group. This means that every trace has to be picked 76/n times (n's are not equal for each group, see table), if every trace of a group is to be picked an equal number of times. Since 76/n is most likely not an integer, we picked every trace at least X times, where X is the closest smaller integer than 76/n (see table). To reach the number of 76 picks, 76 − n × X = Y, see table) traces have to be picked one more time (X + 1 times in total). By picking the traces in the way described here, we ensure that each trace within a group is used at approximately the same frequency for the fit sets. (210 KB PDF) [file pbio.0050311.sg002.pdf]
