# Supplementary material for: Resolving the Fast Kinetics of Cooperative Binding: Ca2+ Buffering by Calretinin
Source: PLoS Biol. 2007 Nov 27;5(11):e311. doi: 10.1371/journal.pbio.0050311 (PMC2229850; doi:10.1371/journal.pbio.0050311)
Supplement: Figure S3 — The figures shown here are identical to Figure 4 (here [A] and [B]) and Figure 5 (here [C] and [D]) in the paper. Depicted here are the fit results for fitting with the new model in red (and pink for apparent K d in [C]). The results for the MWC model (blue symbols in the paper) are omitted for clarity. The black symbols are the fit results when a model for CR was used in which the four cooperative sites are simulated when nH = 1 (no cooperativity). Although fitting the uncaging curves with such a model gives reasonable fits, the results (black symbols) of most of the fitted parameters showed strong deviations when using n H = 1 for the four cooperative sites. This indicated that there is no unique solution to describe CR's Ca2+-binding properties without cooperativity, in line with previous steady-state findings of n H values. (298 KB PDF) [file pbio.0050311.sg003.pdf]

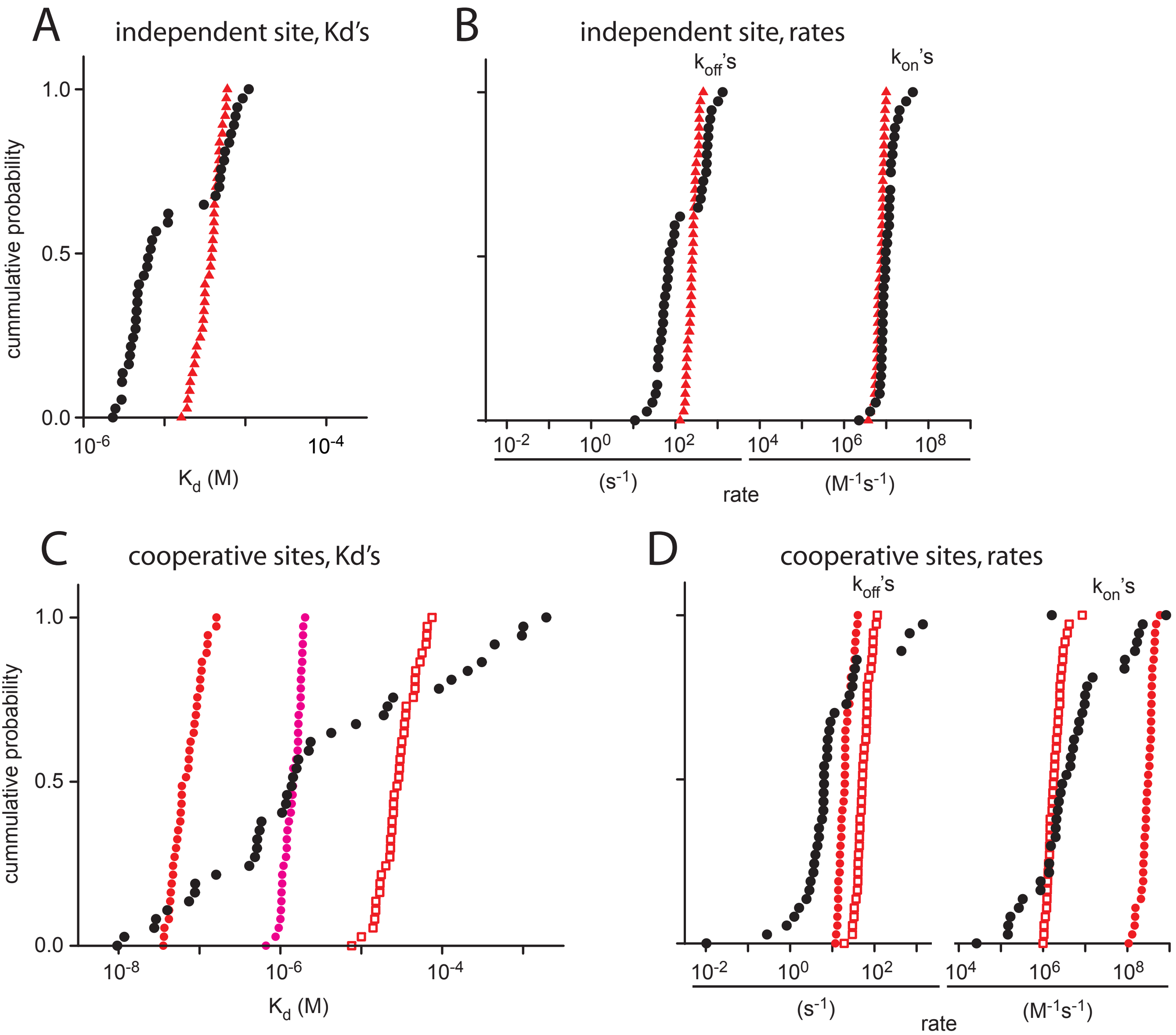
A
independent site, Kd's
cummulative probability
1.0
0.5
0.0
10-6
10-4
Kd (M)
B
independent site, rates
koff's
kon's
10-2
100
102
104
106
108
(s-1)
rate
(M-1s-1)
C
cooperative sites, Kd's
cummulative probability
1.0
0.5
0.0
10-8
10-6
10-4
Kd (M)
D
cooperative sites, rates
koff's
kon's
10-2
100
102
104
106
108
(s-1)
rate
(M-1s-1)
