# Supplementary material for: Resolving the Fast Kinetics of Cooperative Binding: Ca2+ Buffering by Calretinin
Source: PLoS Biol. 2007 Nov 27;5(11):e311. doi: 10.1371/journal.pbio.0050311 (PMC2229850; doi:10.1371/journal.pbio.0050311)
Supplement: Protocol S1 — (1) Supplemental Table 1: Properties of DMn determined from the seven groups of experiments. (2) Supplemental Table 2: KCl-based pCa standard solutions used to calibrate Ca2+-sensistive electrodes and to determine the K d's of various OGB-5N lots. (3) Complete description of the mathematical model (the Berkeley Madonna model) describing the reactions occurring in the experiments represented in a schematic and a mathematical way. (4) Description of the mathematical relationship between the Hill equation and the new and the MWC models via the Adair-Klotz equation for two binding sites. (624 KB DOC) [file pbio.0050311.sd001.doc]

**Supplemental Table 1**

Properties of DM-nitrophen

| K_d (CaDMn)_ | 7.0 | ± | 1.1 | nM |
| --- | --- | --- | --- | --- |
| k_on (CaDMn)_ | 19.8 | ± | 2.5 | μM^-1^s^-1^ |
| k_off (CaDMn)_ | 0.14 | ± | 0.03 | s^-1^ |
| τ_uncaging fast_ | 15.2 | ± | 0.3 | μs |
| fraction τ_uncaging fast_ | 65 | ± | 3 | % |
| τ_uncaging slow_ | 1.9 | ± | 0.1 | ms |
| K_d (CaPP)_ | 4.2 | ± | 0.8 | mM |
| k_on (CaPP)_ | empirically set as k_on (CaDMn)_ | | | |
| k_off (PPDMn)_ | 70 | ± | 6 | ms^-1^ |

**Supplemental Table 2**

***KCl-based pCa standard solutions used to calibrate Ca^2+^-electrodes***

| pCa | Ca^2+^-buffer  (10 mM) | CaCl_2_  (mM) | KOH  (mM) | pH  (measured) | KCl  (mM) | ionic str.  (mM) | calculated [Ca^2+^]_free_ |
| --- | --- | --- | --- | --- | --- | --- | --- |
| ∞ | EDTA | 0.00 | 42 | 7.28 | 78.0 | 120 | 0.00 nM |
| 8.5 | EDTA | 2.80 | 55 | 7.82 | 59.4 | 120 | 3.05 nM |
| 8.0 | EDTA | 2.60 | 45 | 7.29 | 70.8 | 121 | 9.86 nM |
| 7.5 | EDTA | 5.23 | 48 | 7.28 | 62.5 | 121 | 31.6 nM |
| 7.0 | EGTA | 4.93 | 42 | 7.28 | 68.1 | 120 | 101 nM |
| 6.5 | EGTA | 7.17 | 49 | 7.24 | 57.7 | 121 | 316 nM |
| 6.0 | HEEDTA | 2.70 | 36 | 7.50 | 79.6 | 121 | 1.02 μM |
| 5.5 | HEEDTA | 4.10 | 35 | 7.29 | 77.8 | 121 | 3.12 μM |
| 5.0 | HEEDTA | 6.90 | 38 | 7.28 | 68.2 | 120 | 10.1 μM |
| 4.5 | NTA | 2.60 | 37 | 7.42 | 79.8 | 122 | 32.2 μM |
| 4.0 | NTA | 4.53 | 40 | 7.28 | 71.9 | 121 | 101 μM |
| 3.5 | NTA | 7.45 | 40 | 7.29 | 65.1 | 120 | 310 μM |
| 3.0 | -- | 1.00 | 9 | 7.29 | 109.0 | 120 | 1.00 mM |
| 2.5 | -- | 3.16 | 9 | 7.29 | 104.7 | 120 | 3.16 mM |
| 2.0 | -- | 10.00 | 9 | 7.28 | 91.0 | 120 | 10.0 mM |
| 1.5 | -- | 31.62 | 9 | 7.32 | 47.8 | 120 | 31.6 mM |
| 1 | -- | 100.00 | 9 | 7.30 | 0 | 209 | 100 mM |
| 0 | -- | 1000.00 | 9 | 7.30 | 0 | 2009 | 1.00 M |
| All solutions are pH buffered with 20 mM HEPES (4-(2-Hydroxyethyl)piperazine-1-ethylsulfonic acid)  EDTA − Ethylenediamine tetraacetic acid  EGTA − Ethylene glycol-bis(2-aminoethylether)-N,N,N’,N’-tetraacetic acid  HEEDTA − N-(2-Hydroxyethyl)ethylenediamine-N,N’,N’- triacetic acid  NTA − Nitrilo triacetic acid | | | | | | | |
|  | | | | | | | |

**Supplemental Methods**

***The Berkeley Madonna model:***

**Variables**

Values can either be accurately determined (calculated) before the experiment (calc.) or are modeled (simulated) using the model (sim.)

|  | Total Ca^2+^ concentration (calc.), free Ca^2+^ concentration (sim.) and, initial free Ca^2+^ concentration (calc., measured with Ca^2+^-sensitive electrode), respectively |
| --- | --- |
|  | Total DMn concentration (measured with photospectrometer), free DMn concentration (sim.), initial DMn concentration (calc.), concentration of Ca^2+^-DMn complex (sim.), and initial concentration of Ca^2+^-DMn complex (calc). |
| $K_{d(CaDMn)},k_{on(CaDMn)},k_{off(CaDMn)}$ | equilibrium dissociation constant of DMn with Ca^2+^ (fitted in a set of initial experiments with no protein present), association rate constant of DMn with Ca^2+^ (calculated =$k_{off(CaDMn)}/K_{d(CaDMn)}$), and dissociation rate constant of DMn with Ca^2+^ (fitted in a set of initial experiments without protein) |
|  | Photoproduct concentration either formed by uncaging of DMn or CaDMn, initial values are 0, (sim.) and concentration of Ca^2+^-PP complex (sim.). |
|  | Equilibrium dissociation constant of PP with Ca^2+^ , association rate constant of PP with Ca^2+^ (calculated =$k_{off(CaPP)}/K_{d(CaPP)}$) and, dissociation rate constant of PP with Ca^2+^ (values are fitted in a set of initial experiments in the absence of protein) |
| $\alpha, x$ | Fraction of DM-n photolysed (fitted per trace), and the subfraction that photolyzes with the fast time constant (fitted in set of initial experiments*). |
|  | Total DMn concentration and, total CaDMn concentrations that photolyses with the fast time constant (both calc.), respectively |
|  | Total DMn concentration and, total CaDMn concentrations that photolyses with the slow time constant (both calc.), respectively |
| $\tau_{fast}$, $\tau_{slow}$ | Time constant of fast photolysis (fitted in a set of initial experiments) and , time constant of slow photolysis (fitted in a set of initial experiments) |
|  | Flux/rate of fast and slow uncaging of the various photoproducts and complexes of DMn and, CaDMn, respectively (all simulated) |
| $t_{flash}$ | Moment of initiation of photolysis (in these simulations *t=0*) |
| $\left[D\right]_{T},\left[D\right]\left(t\right),\left[CaD\right]\left(t\right),\left[CaD\right]_{t=0}$ | Total dye (Ca^2+^-indicator) concentration = 100 μM OGB-5N, concentration of free dye (sim.), concentration of Ca^2+^-dye complex (sim.) and initial concentration of Ca^2+^-dye complex (calc.) respectively. |
|  | Equilibrium dissociation constant of the dye (measured with Ca^2+^ solutions of known $\left[Ca^{2+}\right]$), association rate constant of the dye (calc., ) and, dissociation rate constant of the dye (measured, derived from relaxation time constant of Ca^2+^-dye complex after Ca^2+^-pulse). |
|  | Fluorescence, initial fluorescence, change in fluorescence compared to initial fluorescence and, ratio $F_{max}/F_{min}$ for the dye, OGB-5N = 10.8 or 40.0 depending on the lot used (measured with Ca^2+^ solutions of known $\left[Ca^{2+}\right]$) |
| $\left[CR\right]_{T}$ | Total CR concentration (measured and fitted) |
| $R,T,B_{V}$ | Binding sites of CR, R represents a cooperative pair in the ‘relaxed’ state, T represents a pair in the ‘tense’ state and B_V_ represents the independent site |
|  | Concentrations of cooperative pairs and individual binding sites that are not occupied with Ca^2+^ (sim.), total concentrations of cooperative pairs (C) and individual binding sites (B_V_) and initial concentrations of these sites that are unoccupied (t=0, calc.) where the cooperative sites are separated in the tense and relaxed state |
| *new model* | Concentration of simulated (pairs of) binding sites and that formed a complex with Ca^2+^ (sim.) and initial concentrations of these complexes (calc), resp. |
| *MWC model* |
| $\begin{matrix} k_{on(V)},k_{off(V)} \\ k_{on(R)},k_{off(R)} \\ k_{on(T)},k_{off(T)} \end{matrix}$ | Association (on) and dissociation (off) rates for the individual binding sites of CR, if modeled as individual sites (and not as cooperative sites) |
| $\begin{matrix} K_{(B_{V})},K_{(R)},K_{(T)} \\ K_{d(B_{V})},K_{d(R)},K_{d(T)} \end{matrix}$ | Association (K) and dissociation (K_d_) constants for the binding sites (at their various states) |
| $L_{(0)},L_{(1)},L_{(2)}$ | Equilibrium constants for transition between R and T state in the MWC model for unoccupied binding sites (T_2_, R_2_), binding sites with one Ca^2+^ bound (CaT_2_, CaR_2_) and with 2 Ca^2+^ bound (Ca_2_T_2_, Ca_2_R_2_), respectively |
| $\begin{matrix} k_{+(L0)},k_{-(L0)} \\ k_{+(L1)},k_{-(L1)} \\ k_{+(L2)},k_{-(L2)} \end{matrix}$ | Rate constants for transition from the T to R state (+) and R to T state (–) in the MWC model for unoccupied binding sites (T_2_, R_2_), binding sites with one Ca^2+^ bound (CaT_2_, CaR_2_) and with 2 Ca^2+^ bound (Ca_2_T_2_, Ca_2_R_2_), respectively |
|  | For the MWC model, total concentration at t=0 of cooperative pairs in the T or R state, respectively |
| $K_{1},K_{2}$ | Macroscopic equilibrium association constant as used in the Adair-Klotz equation (calc.) |

All the values that are a function of time are indicated in the table with (t), however, for clarity this indication is left out in the rest of the descriptions.

**Schematic representation**

***Uncaging and detecting Ca^2+^ in both models***

*Pre flash*

The kinetic reactions for the different fractions before photolysis are:

(S1)

*Flash photolysis of DM-nitrophen*

Of the total DM-nitrophen (DMn) concentration (*[DMn]_T_ = [DMn]+ [CaDMn]*) a certain fraction α photolyzes. The flash energy of the UV laser determines the total proportion (α). Of this fraction α a fraction (*x*) photolyzes rapidly (*[DM]_f_, [CaDMn]_f_*) while a fraction (*1-x*) photolyzes slowly (*[DMn]_s_, [CaDMn]_s_*)

$\left[DMn\right]_{f}=\alpha x\left[DMn\right]$ (S2)

$\left[CaDMn\right]_{f}=\alpha x\left[CaDMn\right]$ (S3)

$\left[DMn\right]_{s}=\alpha\left(1-x\right)\left[DMn\right]$ (S4)

$\left[CaDMn\right]_{s}=\alpha\left(1-x\right)\left[CaDMn\right]$ (S5)

where *x ≤1*.

After the flash the affected DMn molecules transition into photoproducts (PP) of DMn and, CaDMn τ_fast_ and τ_slow_:

$\left[DMn\right]_{f}\xrightarrow{k=\frac{1}{\tau_{fast}}}2\left[PP\right]\xleftarrow{k=\frac{1}{\tau_{slow}}}\left[DMn\right]_{s}$ (S6)

$\left[CaDMn\right]_{f}\xrightarrow{k=\frac{1}{\tau_{fast}}}\left([CaPP]+\left[PP\right]\right)\xleftarrow{k=\frac{1}{\tau_{slow}}}\left[CaDMn\right]_{s}$ (S7)

Photoproducts from Ca^2+^-bound DMn and from free DMn may have different properties as described before ^1^. In our experimental conditions the amount of free DMn is <0.5% and the amount of uncaging is <2%. Consequently, only one in 10^4^ of all DMn molecules will be photolysed into a different PP than the one from CaDMn, making its putative adverse effect negligible. In order to simplify our model, the photoproduct PP from uncaged DMn was considered undistinguishable from PP from uncaged CaDMn leading to the following equilibrium:

(S8)

Note that the on rate of Ca^2+^-binding to PP is the same as that of Ca^2+^-binding to DMn; the effect of uncaging DMn and CaDMn is exerted on the off rate.

*Determining [Ca^2+^] with a Ca^2+^ indicator*

The kinetic reaction for the fluorescent dye (D) is:

(S9)

***Buffering of Ca^2+^ by CR simulated with the new model***

For the new model we simulated CR as one independent Ca^2+^ binding site (B_V_) and two pair of cooperative Ca^2+^ binding sites. To simplify the model it was assumed that both cooperative Ca^2+^ binding sites are identical. So that CR can be described as if it were two independent with the following equilibrium reactions for the cooperative part:

(S10)

(S11)

And for the independent part:

(S12)

where

(S13)

***Buffering of Ca^2+^ by CR simulated with the MWC model***

Cooperativity was also modeled according to the Monod-Wyman-Changeux (MWC) model of cooperativity {MONOD, 1965 MONOD1965 /id} where a cooperative set of binding sites can be either in the ‘relaxed’ state (R) or ‘tense’ state (T). The transition between these states is governed by rate constants k_+_ and k– which are dependent on the occupation level of the cooperative pair:

| 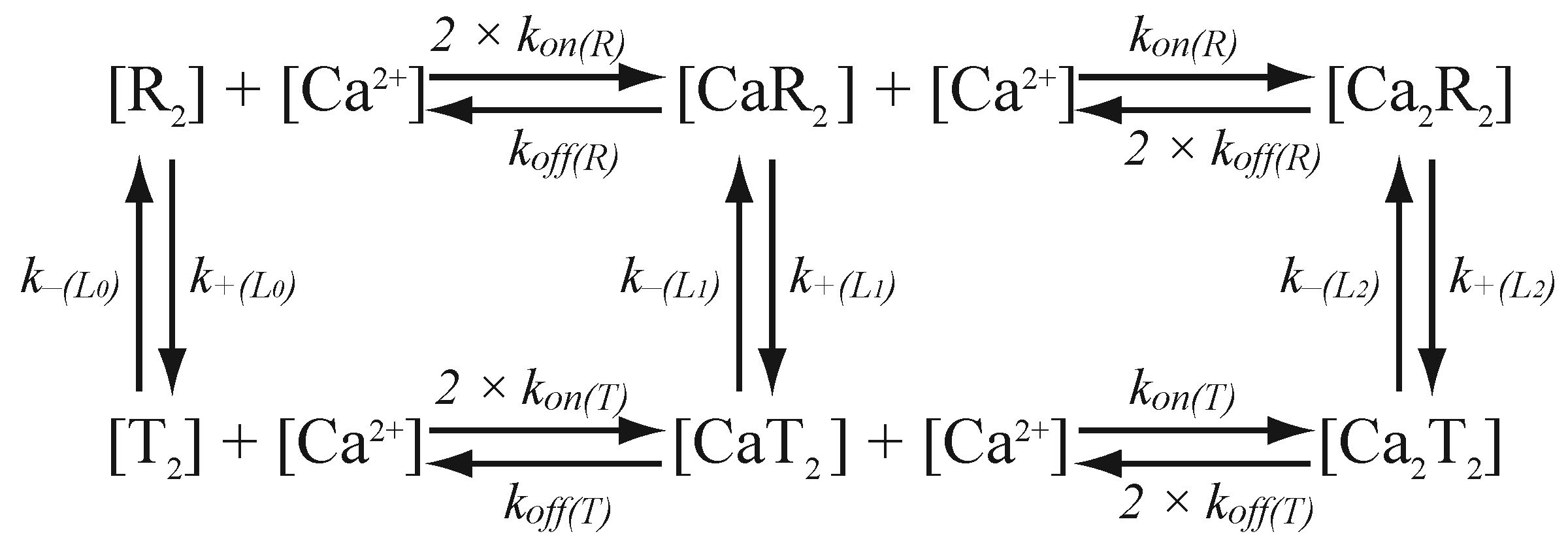 | (S14) |
| --- | --- |

The independent part is identical to the new model and is described by equation 12. Furthermore, equation 13 also holds for the MWC model.

**Mathematical representation**

***Equations for both models***

*For DMn*

$\frac{d\left[CaDMn\right]}{dt}=k_{on(CaDMn)}\cdot\left[Ca^{2+}\right]\cdot\left[DMn\right]-k_{off(CaDMn)}\cdot\left[CaDMn\right]-J_{fast(CaDMn)}-J_{slow(CaDMn)}$ (S15)

$\frac{d\left[DMn\right]}{dt}=-k_{on(CaDMn)}\cdot\left[Ca^{2+}\right]\cdot\left[DMn\right]+k_{off(CaDMn)}\cdot\left[CaDMn\right]-J_{fast(DMn)}-J_{slow(DMn)}$ (S16)

and

$J_{fast(DMn)}=\frac{d\left[DMn\right]_{f}}{dt}=\frac{\left[DMn\right]_{f}}{\tau_{fast}}\cdot T_{flash}$ (S17)

$J_{slow(DMn)}=\frac{d\left[DMn\right]_{s}}{dt}=\frac{\left[DMn\right]_{s}}{\tau_{slow}}\cdot T_{flash}$ (S18)

$J_{fast(CaDMn)}=\frac{d\left[CaDMn\right]_{f}}{dt}=\frac{\left[CaDMn\right]_{f}}{\tau_{fast}}\cdot T_{flash}$ (S19)

$J_{slow(CaDMn)}=\frac{d\left[CaDMn\right]_{s}}{dt}=\frac{\left[CaDMn\right]_{s}}{\tau_{slow}}\cdot T_{flash}$ (S20)

where *T_flash_ = 0* if *t < t_flash_* (S21)

and *T_flash_ = 1* if *t ≥ t_flash_* (S22)

*For the photoproduct:*

$\frac{d\left[CaPP\right]}{dt}=k_{on(CaDMn)}\cdot\left[Ca^{2+}\right]\cdot\left[PP\right]-k_{off(CaPP)}\cdot\left[CaPP\right]+J_{fast(CaDMn)}+J_{slow(CaDMn)}$ (S23)

$\begin{matrix} \frac{d\left[PP\right]}{dt}=-k_{on(CaDMn)}\cdot\left[Ca^{2+}\right]\cdot\left[PP\right]+k_{off(CaPP)}\cdot\left[CaPP\right]+2J_{fast(DMn)} \\ +2J_{slow(DMn)}+\cdot J_{fast(CaDMn)}+\cdot J_{slow(CaDMn)} \end{matrix}$ (S24)

*For the dye*

$\frac{d\left[CaD\right]}{dt}=k_{on(D)}\cdot\left[Ca^{2+}\right]\cdot\left[D\right]-k_{off(D)}\cdot\left[CaD\right]$ (S25)

$\left[D\right]=\left[D\right]_{T}-\left[CaD\right]$ (S26)

***Equations for CR, modeled with the new model***

$\frac{d\left[CaTR\right]}{dt}=2k_{on(T)}\cdot\left[Ca^{2+}\right]\cdot\left[T_{2}\right]-k_{off(T)}\cdot\left[CaTR\right]$ (S27)

$\frac{d\left[Ca_{2}R_{2}\right]}{dt}=k_{on(R)}\cdot\left[Ca^{2+}\right]\cdot\left[CaTR\right]-2k_{off(R)}\cdot\left[Ca_{2}R_{2}\right]$ (S28)

$\left[T_{2}\right]=\left[C\right]_{T}-\left[CaTR\right]-\left[Ca_{2}R_{2}\right]$ (S29)

$\frac{d\left[CaB_{V}\right]}{dt}=k_{on(V)}\cdot\left[Ca^{2+}\right]\cdot\left[B_{V}\right]-k_{off(V)}\cdot\left[CaB_{V}\right]$ (S30)

$\left[B_{V}\right]=\left[B_{V}\right]_{T}-\left[CaB_{V}\right]$ (S31)

$\left[Ca^{2+}\right]=\left[Ca^{2+}\right]_{T}-\left[CaDMn\right]-\left[CaPP\right]-\left[CaD\right]-\left[CaTR\right]-2\left[Ca_{2}R_{2}\right]-\left[CaB_{V}\right]$ (S32)

and

$\left[Ca^{2+}\right]_{T}=\left[Ca^{2+}\right]_{t=0}+\left[CaDMn\right]_{t=0}+\left[CaD\right]_{t=0}+\left[CaTR\right]_{t=0}+2\left[Ca_{2}R_{2}\right]_{t=0}+\left[CaB_{V}\right]_{t=0}$ (S33)

With initial conditions:

$\left[CaTR\right]_{t=0}=\frac{K_{1}\left[Ca^{2+}\right]_{t=0}}{1+K_{1}\left[Ca^{2+}\right]_{t=0}+K_{1}K_{2}\left[Ca^{2+}\right]_{t=0}^{2}}\cdot\left[C\right]_{T}$ (S34)

$\left[Ca_{2}R_{2}\right]_{t=0}=\frac{K_{1}K_{2}\left[Ca^{2+}\right]_{t=0}^{2}}{1+K_{1}\left[Ca^{2+}\right]_{t=0}+K_{1}K_{2}\left[Ca^{2+}\right]_{t=0}^{2}}\cdot\left[C\right]_{T}$ (S35)

$\left[T_{2}\right]_{t=0}=\left[C\right]_{T}-\left[CaTR\right]_{t=0}-\left[Ca_{2}R_{2}\right]_{t=0}$ (S36)

$\left[CaB_{V}\right]_{t=0}=\frac{\left[Ca^{2+}\right]_{t=0}}{K_{d(V)}+\left[Ca^{2+}\right]_{t=0}}\cdot\left[B_{V}\right]_{T}$ (S37)

where

$K_{1}=2\times K_{(T)}$ (S38)

$K_{1}K_{2}=K_{(T)}K_{(R)}$ (S39)

$K_{x}=\frac{k_{on(x)}}{k_{off(x)}}=\frac{1}{K_{d(x)}}$ (S40)

and

$\left[C\right]_{T}=2\left[CR\right]_{T}$ (S41)

$\left[B_{V}\right]_{T}=\left[CR\right]_{T}$ (S42)

***Equations for CR, modeled with the MWC model***

$\frac{d\left[T_{2}\right]}{dt}=-2k_{on(T)}\cdot\left[Ca^{2+}\right]\cdot\left[T_{2}\right]+k_{off(T)}\cdot\left[CaT_{2}\right]-k_{-(L0)}\cdot\left[T_{2}\right]+k_{+(L0)}\cdot\left[R_{2}\right]$ (S43)

$\begin{matrix} \frac{d\left[CaT_{2}\right]}{dt}=2k_{on(T)}\cdot\left[Ca^{2+}\right]\cdot\left[T_{2}\right]-k_{off(T)}\cdot\left[CaT_{2}\right]-k_{on(T)}\cdot\left[Ca^{2+}\right]\cdot\left[CaT_{2}\right]+2k_{off(T)}\cdot\left[Ca_{2}T_{2}\right] \\ -k_{-(L1)}\cdot\left[CaT_{2}\right]+k_{+(L1)}\cdot\left[CaR_{2}\right] \end{matrix}$ (S44)

$\frac{d\left[Ca_{2}T_{2}\right]}{dt}=k_{on(T)}\cdot\left[Ca^{2+}\right]\cdot\left[CaT_{2}\right]-2k_{off(T)}\cdot\left[Ca_{2}T_{2}\right]-k_{-(L2)}\cdot\left[Ca_{2}T_{2}\right]+k_{+(L2)}\cdot\left[Ca_{2}R_{2}\right]$ (S45)

$\frac{d\left[R_{2}\right]}{dt}=-2k_{on(R)}\cdot\left[Ca^{2+}\right]\cdot\left[R_{2}\right]+k_{off(R)}\cdot\left[CaR_{2}\right]+k_{-(L0)}\cdot\left[T_{2}\right]-k_{+(L0)}\cdot\left[R_{2}\right]$ (S46)

$\begin{matrix} \frac{d\left[CaR_{2}\right]}{dt}=2k_{on(R)}\cdot\left[Ca^{2+}\right]\cdot\left[R_{2}\right]-k_{off(R)}\cdot\left[CaR_{2}\right]-k_{on(R)}\cdot\left[Ca^{2+}\right]\cdot\left[CaR_{2}\right]+2k_{off(R)}\cdot\left[Ca_{2}R_{2}\right] \\ +k_{-(L1)}\cdot\left[CaT_{2}\right]-k_{+(L1)}\cdot\left[CaR_{2}\right] \end{matrix}$ (S47)

$\frac{d\left[Ca_{2}R_{2}\right]}{dt}=k_{on(R)}\cdot\left[Ca^{2+}\right]\cdot\left[CaR_{2}\right]-2k_{off(R)}\cdot\left[Ca_{2}R_{2}\right]+k_{-(L2)}\cdot\left[Ca_{2}T_{2}\right]-k_{+(L2)}\cdot\left[Ca_{2}R_{2}\right]$ (S48)

$\frac{d\left[CaB_{V}\right]}{dt}=k_{on(V)}\cdot\left[Ca^{2+}\right]\cdot\left[B_{V}\right]-k_{off(V)}\cdot\left[CaB_{V}\right]$ (S49)

$\left[B_{V}\right]=\left[B_{V}\right]_{T}-\left[CaB_{V}\right]$ (S50)

$\begin{matrix} \left[Ca^{2+}\right]=\left[Ca^{2+}\right]_{T}-\left[CaDMn\right]-\left[CaPP\right]-\left[CaD\right]-\left[CaT_{2}\right]-2\left[Ca_{2}T_{2}\right] \\ -\left[CaR_{2}\right]-2\left[Ca_{2}R_{2}\right]-\left[CaB_{V}\right] \end{matrix}$ (S51)

and

$\begin{matrix} \left[Ca^{2+}\right]_{T}=\left[Ca^{2+}\right]_{t=0}+\left[CaDMn\right]_{t=0}+\left[CaD\right]_{t=0}+\left[CaT_{2}\right]_{t=0}+2\left[Ca_{2}T_{2}\right]_{t=0} \\ +\left[CaR_{2}\right]_{t=0}+2\left[Ca_{2}R_{2}\right]_{t=0}+\left[CaB_{V}\right]_{t=0} \end{matrix}$ (S52)

With initial conditions:

(S53)

(S54)

(S55)

(S56)

(S57)

(S58)

(S59)

where $L_{1}=\frac{\left[CaT_{2}\right]}{\left[CaR_{2}\right]}=\frac{L_{0}\left[R_{2}\right]\cdot\left[Ca^{2+}\right]\cdot K_{T}}{\left[R_{2}\right]\cdot\left[Ca^{2+}\right]\cdot K_{R}}=L_{0}\frac{K_{T}}{K_{R}}$ (S60)

(S61)

where $L_{2}=\frac{\left[Ca_{2}T_{2}\right]}{\left[Ca_{2}R_{2}\right]}=\frac{L_{0}\left(\left[R_{2}\right]\cdot\left[Ca^{2+}\right]\cdot K_{T}\right)^{2}}{\left(\left[R_{2}\right]\cdot\left[Ca^{2+}\right]\cdot K_{R}\right)^{2}}=L_{0}\left(\frac{K_{T}}{K_{R}}\right)^{2}$ (S62)

and for MWC model also equations 37, 41 and 42 have to be considered.

***Other initial conditions for both models***

$\left[DMn\right]_{t=0}=\left[DMn\right]_{T}-\left[CaDMn\right]_{t=0}-\left[MgDMn\right]_{t=0}$ (S63)

$\left[CaDMn\right]_{t=0}=\frac{\left[Ca^{2+}\right]_{t=0}}{K_{d(CaDMn)}+\left[Ca^{2+}\right]_{t=0}}\cdot\left[DMn\right]_{T}$ (S64)

$\left[CaD\right]_{t=0}=\frac{\left[Ca^{2+}\right]_{t=0}}{K_{d(CaD)}+\left[Ca^{2+}\right]_{t=0}}\cdot\left[D\right]_{T}$ (S65)

***Model output for both models***

$\frac{\Delta F}{F}=\frac{\Delta F\left(t\right)}{F_{t=0}}=\frac{\left[CaD\right]\cdot\left(F_{ratio}-1\right)+\left[D\right]_{T}}{\left[CaD\right]_{t=0}\cdot\left(F_{ratio}-1\right)+\left[D\right]_{T}}$ (S66)

**From Hill to Adair-Klotz to Microscopic Binding Constants**

To aid the choice of starting values for the fits, the cooperative part of the model was set up so that *k_on(Co-0)_*, *k_on(Co-1)_*, the *apparent K_d_* (*K_d(app)_*) for the pairs and the Hill number *n_H_*, could be fitted. This was achieved adding a calulation step that determined *K_d(Co-0)_* and *K_d(Co-1)_* from the latter two parameters. The average number of binding sites occupied by Ca^2+^ (ν) is according to the Hill equation:

$\nu=f\left(\left[Ca^{2+}\right]\right)=2\times\frac{\left[Ca^{2+}\right]^{n_{H}}}{\left[Ca^{2+}\right]^{n_{H}}+K_{d(app)}^{n_{H}} }$ (S67)

For the Adair-Klotz approach this would correspond to:

$\nu=g\left(\left[Ca^{2+}\right]\right)=\frac{K_{1}\left[Ca^{2+}\right]+2K_{1}K_{2}\left[Ca^{2+}\right]^{2}}{1+K_{1}\left[Ca^{2+}\right]+K_{1}K_{2}\left[Ca^{2+}\right]^{2}}$ (S68)

It can be shown that:

$n_{H}\neq1\to f\left(\left[Ca^{2+}\right]\right)\neq g\left(\left[Ca^{2+}\right]\right)$ (S69)

However, both equations are widely accepted ways to describe equilibrium binding and are indeed very similar. Both functions are point symmetrical around the half maximal value (in this case ν=1) where [Ca^2+^] = *K_d(app)_*. Therefore, the basic sigmoid shape of the binding curve can be described by the value of *K_d(app)_* and the slope at this point, where [Ca^2+^] = *K_d(app)_*, as it is explicitly in *n_H_* in the case of the Hill model. For the closest correlation between the Hill and Adair-Klotz model both should have the half maximal point at the same [Ca^2+^], so that:

$\left[Ca^{2+}\right]=K_{d(app)}\to f\left(K_{d(app)}\right)=g\left(K_{d(app)}\right)=1$ (S70)

thus

$g\left(K_{d(app)}\right)=1\to\frac{K_{1}K_{d(app)}+2K_{1}K_{2}K_{d(app)}^{2}}{1+K_{1}K_{d(app)}+K_{1}K_{2}K_{d(app)}^{2}}=1$ (S71)

Furthermore, the slope at this point of symmetry should be the same, so that:

$f'\left(K_{d}\right)=g'\left(K_{d}\right)$ (S72)

which is

$\frac{K_{1}+4K_{d(app)}K_{1}K_{2}}{1+K_{d(app)}K_{1}+K_{d(app)}^{2}K_{1}K_{2}}-\frac{\left(K_{1}+2K_{d(app)}K_{1}K_{2}\right)\left(K_{d}K_{1}+2K_{d(app)}^{2}K_{1}K_{2}\right)}{\left(1+K_{d(app)}K_{1}+K_{d(app)}^{2}K_{1}K_{2}\right)^{2}}=\frac{n_{H}}{2K_{d(app)}}$ (S73)

From equations 71 and 73 we derive:

$K_{1}=\frac{4-2n_{H}}{K_{d(app)}n_{H}}$ (S74)

$K_{2}=\frac{n_{H}}{4K_{d(app)}-2n_{H}}$ (S75)

$K_{d(app)}=\frac{1}{\sqrt{K_{1}K_{2}}}$ (S76)

$n_{H}=\frac{4}{2+\sqrt{\frac{K_{1}}{K_{2}}}}$ (S77)

***For the new model***

To express the relation of *K_d(app)_* and *n_H_* to microscopic binding constants in terms of the new model we derive from equations 38-40 and 74-77:

$K_{d(app)}=\sqrt{K_{d(T)}K_{d(R)}}$ (S78)

$n_{H}=\frac{2}{1+\sqrt{\frac{K_{d(R)}}{K_{d(T)}}}}$ (S79)

and

(S80)

$K_{d(R)}=\frac{2K_{d(app)}-n_{H}}{n_{H}}$ (S81)

***For the MWC model***

To express the MWC model in terms of the Adair-Klotz model we have to consider that:

(S82)

(S83)

where

$\alpha=\frac{K_{T}}{K_{R}}$ (S84)

From equations 76, 77 and 82-84 we derive:

$K_{d(app)}=\frac{1}{K_{R}\sqrt{\frac{\alpha+L_{0}}{1+L_{0}}\frac{\alpha^{2}+L_{0}}{\alpha+L_{0}}}}$ (S85)

$n_{H}=\frac{4}{2+\sqrt{\frac{\left(\alpha+L_{0}\right)^{2}}{\left(1+L_{0}\right)\left(\alpha^{2}+L_{0}\right)}}}$ (S86)
